# Patient and provider perspectives on polygenic risk scores: implications for clinical reporting and utilization

**DOI:** 10.1186/s13073-022-01117-8

**Published:** 2022-10-07

**Authors:** Anna C. F. Lewis, Emma F. Perez, Anya E. R. Prince, Hana R. Flaxman, Lizbeth Gomez, Deanna G. Brockman, Paulette D. Chandler, Benjamin J. Kerman, Matthew S. Lebo, Jordan W. Smoller, Scott T. Weiss, Carrie L. Blout Zawatksy, James B. Meigs, Robert C. Green, Jason L. Vassy, Elizabeth W. Karlson

**Affiliations:** 1 E.J. Safra Center for Ethics, Harvard University, Cambridge, USA; 2 Department of Medicine, Brigham and Women’s Hospital, Boston, MA USA; 3 Mass General Brigham Personalized Medicine, Boston, MA USA; 4 College of Law, University of Iowa, Iowa City, IA USA; 5 Weill Cornell Medical College, New York City, NY USA; 6 Color Health, Burlingame, CA USA; 7 Department of Pathology, Brigham and Women’s Hospital, Boston, MA USA; 8 Harvard Medical School, Boston, MA USA; 9 Program in Medical and Population Genetics, Broad Institute of MIT and Harvard, Cambridge, MA USA; 10 Center for Genomic Medicine, Massachusetts General Hospital, Boston, MA USA; 11 Center for Precision Psychiatry, Massachusetts General Hospital, Boston, MA USA; 12 Stanley Center for Psychiatric Research, Broad Institute of MIT and Harvard, Cambridge, MA USA; 13 Channing Division of Network Medicine, Boston, MA USA; 14 Population Precision Health, Ariadne Labs, Boston, MA USA; 15 The MGH Institute of Health Professions, Boston, MA USA; 16 Division of General Internal Medicine, Massachusetts General Hospital, Boston, MA USA; 17 Mass General Brigham Personalized Medicine, Cambridge, MA USA; 18 Veterans Affairs Boston Healthcare System, Boston, MA USA; 19 Division of General Internal Medicine and Primary Care, Brigham and Women’s Hospital, Boston, MA USA

**Keywords:** Polygenic risk scores, Report design, Preventative medicine, Personalized medicine, Qualitative semi-structured interviews

## Abstract

**Background:**

Polygenic risk scores (PRS), which offer information about genomic risk for common diseases, have been proposed for clinical implementation. The ways in which PRS information may influence a patient’s health trajectory depend on how both the patient and their primary care provider (PCP) interpret and act on PRS information. We aimed to probe patient and PCP responses to PRS clinical reporting choices

**Methods:**

Qualitative semi-structured interviews of both patients (*N*=25) and PCPs (*N*=21) exploring responses to mock PRS clinical reports of two different designs: binary and continuous representations of PRS.

**Results:**

Many patients did not understand the numbers representing risk, with high numeracy patients being the exception. However, all the patients still understood a key takeaway that they should ask their PCP about actions to lower their disease risk. PCPs described a diverse range of heuristics they would use to interpret and act on PRS information. Three separate use cases for PRS emerged: to aid in gray-area clinical decision-making, to encourage patients to do what PCPs think patients should be doing anyway (such as exercising regularly), and to identify previously unrecognized high-risk patients. PCPs indicated that receiving “below average risk” information could be both beneficial and potentially harmful, depending on the use case. For “increased risk” patients, PCPs were favorable towards integrating PRS information into their practice, though some would only act in the presence of evidence-based guidelines. PCPs describe the report as more than a way to convey information, viewing it as something to structure the whole interaction with the patient. Both patients and PCPs preferred the continuous over the binary representation of PRS (23/25 and 17/21, respectively). We offer recommendations for the developers of PRS to consider for PRS clinical report design in the light of these patient and PCP viewpoints.

**Conclusions:**

PCPs saw PRS information as a natural extension of their current practice. The most pressing gap for PRS implementation is evidence for clinical utility. Careful clinical report design can help ensure that benefits are realized and harms are minimized.

**Supplementary Information:**

The online version contains supplementary material available at 10.1186/s13073-022-01117-8.

## Background

Polygenic risk scores (PRS) aggregate the impact of many genetic variants associated with a condition to give an aggregated indication of genomic risk for that condition. Utilizing genome-wide association study (GWAS) data, PRS are validated in independent data sets to estimate the strength of the score as a predictor of the condition. Some PRS are as predictive of a condition as monogenic risk factors already in clinical use, which has prompted calls for their clinical adoption [1]. PRS are currently available from some genetic testing companies, and can be ordered by a physician or as a direct-to-consumer test [2, 3]. Their clinical use is also being assessed in clinical trials and implementation studies [4–7]. There are several known barriers to the clinical adoption of PRS, including the current limited evidence for their clinical utility, and the known differential predictive performance of the scores in different population groups [8, 9]. Despite these concerns, early evidence suggests that there may be some clinical utility to sharing PRS in the preventive health setting [10–12]. Because we currently have very limited information about the clinical impact of sharing PRS, evidence-based guidelines supporting the use of PRS do not yet exist.

For a PRS to be reported to an individual patient, either in the context of the clinic or clinical research, choices must be made about how to convey the associated risk information on a clinical report. However, there are no published guidelines for how this should be done. This stands in contrast with the reporting of genetic information associated with single high penetrance mutations, where clear guidelines have been established for every aspect of clinical reporting [13–15]. Unsurprisingly, PRS reports generated to date are very divergent, reflecting the fact that there are multiple key decisions that must be made [16]. Early evidence suggests that understanding of PRS reports can be very limited [17].

How risk information is communicated greatly impacts a patient’s understanding and perception of risk, and how the primary care provider (PCP) interprets the data and/or communicates it to the patient [18, 19]. How information is presented on clinical reports will hence impact the balance of benefits and potential harms of the clinical use of PRS. Moreover, interventions that rely on reporting personalized health information can contribute to inequities, for example, if information is communicated in such a way that it is better understood or more effectively used by those from socioeconomically advantaged groups [20]. Different risk presentation strategies can also maximize different end points; for example, the same strategy may not simultaneously maximize the accuracy of interpretation and the intention to change behavior [19, 21]. It is not always the case that incorporating all the relevant information leads to the most accurate judgments [22]. These issues in risk communication extend far beyond the clinical reporting of PRS and need to be considered against the backdrop of widespread mis-estimation of risk levels by medical practitioners [23].

To determine the likely impact of clinical reporting choices so as to maximize the benefits and minimize the harms of their potential clinical deployment, it is necessary to understand the views of both patients and their providers. In the case of PRS, because they generally report on risk for the common conditions that are initially detected and managed by primary care providers (PCPs), the views of PCPs are particularly relevant. We are aware of two studies reporting stakeholder views on PRS clinical report design, both on patient perspectives: a user-experience study [16] and a focus group study [24]. This study adds to the literature by probing both patient and PCP responses to clinical PRS reporting choices, which can then inform future implementation considerations.

## Methods

In our study, for both PCPs and patients, we aimed to assess their interpretations, reactions, and preferences for different PRS clinical reporting choices, including their willingness to act on the information in the reports. We also aimed to understand how the reports would be used in a discussion between patients and their PCPs. We hence asked about the questions patients would have for their PCP, and the PCPs’ anticipations for the conversation they would have with a patient. We also aimed to assess the more general attitudes of PCPs towards the incorporation of PRS into their practice. In addition to contributing to the literature on the clinical use of PRS, our study sought to inform the clinical report design used by eMERGE IV, a multi-site implementation study that is returning health risk reports featuring PRS, monogenic results, family history, and clinical risk factors for eleven different conditions to 25,000 Americans [25].

### Mock report design

We constructed two mock clinical report designs, one using a binary representation and one a continuous representation of PRS results. We showed participants two different versions of the binary report, one indicating “high risk” and one “not identified as at high risk.” These reports indicated the threshold considered “high risk” as the 95th percentile and the odds ratio(s) at this percentile. The continuous report design gave the percentile and the associated odds ratio(s). This report design utilized a bell curve graphic, indicating where this individual fell on the bell curve. We showed participants one continuous report indicating the individual was at the 99th percentile, and another indicating they were at the 75th percentile. We chose to not include an absolute risk representation because only two of the eleven conditions reported in eMERGE IV will report an absolute risk (breast cancer and coronary heart disease), while the remaining nine will report odds ratios. Each report also gave the prevalence of the condition in the overall population. The report designs were slightly different for the patients than for the PCPs. The PCPs reviewed mock reports for a PRS for prostate cancer, chosen because current guidelines neither advocate strongly for or against screening in the general population. The patients instead reviewed a mock report for “disease x,” because we aimed to probe reactions to receiving disease risk information in general, rather than for a specific condition.

For the patient reports, a single odds ratio was given. For the PCP reports, the odds ratio information was preceded by the sentence “Those at high risk have increased odds of developing prostate cancer that varies by ancestry.” A set of ranges of odds ratios followed, one for each of Asian ancestry, African ancestry, European ancestry, and Latino/a/x ancestry. Each report also contained a limitations section. For the patient report, this included a sentence that “the majority of existing data used to calculate polygenic risk scores comes from individuals of European ancestry,” and for the PCP report, “Although the polygenic score predicts risk in all ancestries, the scores have been best validated in individuals of European ancestry.” These population groups were based on those used in eMERGE IV (“African, European, East Asian and Hispanic/Latino descent”).

Our initial report designs were informed by existing PRS reports [7, 16, 26, 27], and the known constraints of eMERGE IV, such as that most scores would be reported via odds ratios, and that these would be calculated in four populations [28]. The initial designs were shared with all members of the multidisciplinary eMERGE IV MGB site team — including several PCPs and bioethicists — for their input. The designs were iterated upon based on this feedback. The reports are available within the interview guides provided in Additional file 1.

### Recruitment

For the recruitment of patients, we utilized the Mass General Brigham (MGB) Biobank [29]. Approximately 130,000 individuals are enrolled in this biobank, and the majority have given their consent to be contacted about additional research opportunities. A cohort of patients was generated by the MGB Biobank team for the study team to recontact. Patients that were deceased, who consented through a surrogate, or who did not consent to recontact were removed. We sought to recruit five individuals self-identifying as Asian, Black, Hispanic/Latinx, and White, all English-speaking, and five Spanish-speaking individuals (25 total). MGB Biobank participants were contacted by letter and phone, with the contacts prioritized to ensure the recruitment targets were met. For the recruitment of PCPs, we used email to contact Doctors of Medicine (MDs), Nurse Practitioners (NPs), and Physician Assistants (PAs) practicing at clinics associated with Brigham and Women’s Hospital and at Massachusetts General Hospital (256 in total). We stopped recruiting after enrolling 21 PCPs. Sample sizes were designed to achieve data saturation, i.e., to be large enough such that new samples did not yield new themes [30].

### Data collection

Prior to the interviews, participants for the patient interviews were asked to complete three short surveys: a 19-item survey assessing genetic literacy (the University of North Carolina Genomic Knowledge Scale, UNC-GKS) [31]; an 8-item instrument asking participants to self-assess their numeracy (the Subjective Numeracy Scale, SNS) [32]; and a 3-item scale assessing health literacy (the Short Test of Functional Health Literacy in Adults, STOHFLA) [33]. Details of how these scales are scored are given in Additional file 1. At the start of the interviews, patient participants were educated about PRS utilizing an educational resource assembled and hosted at the Broad Institute [34]. In addition to explaining what a PRS is, this resource highlights that “DNA is not destiny” and that additional factors influence risk. Both English and Spanish versions of the clinical reports, surveys, and educational materials were available. The patient participants were shown a series of reports and asked how they interpreted them, whether they had any questions about them, and what questions they would have for their PCP. They were also asked similar questions about the limitations section. They were directly asked for their preferences between the binary and continuous designs. Finally, they were asked: ”If you were asked if you would like to receive a polygenic risk score result, would you still like to receive this information if the results may be inexact for your ancestry?” Interviews lasted about 45 minutes and patients were compensated $25 for their time.

The PCP interviews started with a very brief synopsis describing PRS calculations. PCPs were asked about their prior exposure to genetics and to PRS, and about their attitudes to prostate-specific antigen (PSA) screening. For each report design they were asked about their potential interpretation, any questions they might have, their comfort level discussing the report with their patient, and the actions they would consider in light of the information. They were also asked about their understanding of the limitations section, and how they would talk about this report with a patient of non-European ancestry. They were asked for their preferences between the continuous and binary report design, and about their perspective on the use of a threshold to identify those at increased risk. Finally, they were asked about the barriers and benefits they saw for the integration of this type of report into primary care, the conditions under which they would refer to a genetic counselor, and what PCP education, if any, they would consider necessary and practical. At the end of the interviews, PCPs were asked some demographic information. Interviews lasted 60 minutes and PCPs were compensated $200 for their time. See Additional file 1 for the semi-structured interview guides.

### Analysis

All interviews were conducted and recorded on Zoom and the audio was transcribed using NVivo. The transcripts were cleaned (to make the text legible without changing the meaning of what was said) and anonymized (to ensure interviewees could not be traced) prior to analysis. A group of study investigators met to construct an initial coding scheme largely based on the interview guides but also incorporating insights from relevant prior literature. This coding scheme, which was designed to be used for both sets of interviews (patient and PCP), was iterated on after review of several transcripts from both sets of interviews. Two initial reviewers coded each transcript based on this initial coding scheme. Intercoder agreement was established based on comparison of several transcripts. For many of the codes, in particular for the patient interviews, sections of text that could be coded as binary were categorized by the reviewers, for example, whether or not a participant mentioned an aspect of the report design, or what their preference was for a reporting choice. For these cases where we were able to categorize answers, and hence provide counts of responses, two coders independently assessed the coded sections, and any disagreements were discussed and resolved via reference to the original transcripts. Such disagreements were very rare and typically resulted from a lack of clarity in our categorization scheme. For the patient interviews, no additional themes emerged beyond those in the code book. For the PCP interviews, additional themes emerged during the analysis of the coding and notes. Throughout the coding and review process, team members met frequently to ensure consistency and discuss analysis.

## Results

### Patient interviews

#### Patient understanding of and reaction to risk information

Patient baseline and demographic information are given in Additional file 1: Table S1. As per our recruitment strategy, five individuals self-identified as non-Hispanic Asian, five as non-Hispanic Black, five as non-Hispanic White, and ten as Hispanic White, five of whom were Spanish speakers. Five (20%) had low genetic literacy. For self-assessed numeracy, three (12%) were low, thirteen (52%) average, and nine (36%) high. Nine (36%) had inadequate health literacy.

Patients selectively engaged with the different types of information presented. The percentile risk rank included in the mock report was the number most engaged with (21/25), but the majority were confused by it (12/21), including two high numeracy patients. Many of these mistook percentile for percent chance. For example: “He‘s almost at a full whole risk. Ninety-nine percentile is almost at one hundred, so it’s like you’re one percent away from being completely at all risk of getting it. Doesn’t matter what age it is, you’re going to get it, that’s the thing.” There were other forms of confusion, for example: “That the person in the evaluation, those that have 95, have the least risk. And those with the least percentage, they have the most risk.” A minority (9/25) understood the percentile information, of which seven were high numeracy patients. Most patients referred to the odds ratio (15/25), the majority of whom were confused by it (9/15), including two with high numeracy. All of the patients who understood the odds ratios had high numeracy. Most patients interacted with the prevalence information (14/25), about half of whom were confused by it (6/14). For example, some thought the prevalence was their individual result. All of those who understood it had high numeracy. A common misinterpretation was to interpret “not high risk” as “low risk.” Also, in the context of the binary report, a common misinterpretation was to view the threshold as representing their own risk.

Additional information was desired; many wanted to see the inclusion of absolute risk information, and an integrated score: “I want the full assessment… and you’re only giving me the genetic score, which is necessary but not sufficient for a real assessment of my risk”. Two patients wanted to understand the genetic attributable risk, i.e., how much of the overall risk is captured by genetics, and by this score specifically: “I would want to say that it’s [patient’s risk] high, but not really, since there’s so many other factors that can contribute to getting a disease. And it’s not just your genetics. So it does come to this question in my head, how important is it really if it’s just like a small factor and it's not really like the only thing that means you're going to get the disease.”

When asked how they would interpret a fictional patient’s risk, many patients did not engage with the limitations section until explicitly prompted to do so. Most did not engage with the ancestry limitation. For those that did, many of them thought it should explain implications for patients: “it’s just it’s not really fully explaining, like, the implications”. Some did not understand: “Well, it says European ancestry. ... and I don’t understand when they say European, are they talking in Spain, Portugal?”

Of those patients asked whether they would “still like to receive this information if the results may be inexact for your ancestry?” (16/25) only a small handful (3/16) said they would not want it. There were no discernible patterns by the patients’ self-identified race/ethnicity.

#### Binary/continuous preference

Almost all (23/25) of the patients preferred the continuous report design. The few voices in favor of the binary report appreciated how direct the information was. In contrast to this, one patient expressed a common sentiment in reaction to the binary reports: the high binary “just made me feel like I have the disease, whatever it is” and the not high binary report “just doesn't give enough information.”

In their preference for the continuous report design, knowing their exact risk number was the most common reason given for their preferences. Patients expressed that not knowing their own risk number would leave them with questions, make them uncomfortable, and make them feel less empowered to alter their chances of developing a condition. They also wondered why the information was not being shared with them: “I would like to know my polygenic risk… Ignorance is bliss. Great. But knowledge is power. So I need to know my risk. If I know my risk, I can alter it, change it, and I can feel better about this thing. At least I can do something.” Another said, “I mean, the threshold here is like all I know is I’m not in the top 2 percent, it doesn’t really, what if I’m in the ninety seventh percent. ... it seems like a very narrow definition for having a high polygenic risk factor. But if you have that data, why not give it to me?”

Patients also appreciated use of the graph in the continuous report design, as an aid to understanding the risk information: “If I visually have a picture to match what I’m reading, it helps, just me personally, me, it helps me see visually what I would- what they’re trying to tell me…. seeing and knowing are two things that are always going to go hand in hand.”

#### Emotional reaction

A common theme that emerged was anticipated or imagined emotional reaction to the reports. A substantial minority (8/25) had an emotional reaction to the high binary report, and likewise (though fewer, 4/25) for the 99th percentile continuous report. This was often linked to a sense of genetic determinism, that they are going to develop the condition: “I mean, it gets to the point, but, me being the person that I am and not knowing too many big words, a lot of this would kind of scare me... just seeing the ‘high risk’, I probably would think that ‘Oh my God, I’m going to get cancer’.” And “it tells you for real, tells you to start getting ready. But I would die, it's very severe. Very alarming, very severe, It would make me worried.” We note that while both the educational materials and the reports themselves contained information designed to counter genetic determinism, our results suggest that this information was not effective at addressing these attitudes, suggesting that these reactions may be hard to dispel. Some had an emotional frame of being relaxed or not alarmed at the not high binary and the lower continuous report.

#### Questions for PCPs

When patients were asked “what questions would you have for your provider?,” all would ask what they could do to lower their risk. About half would ask for help interpreting the reports. Only a few other types of questions were raised, including what would happen if they develop the condition, for example, age of onset and disease trajectory.

### Primary care provider (PCP) interviews

PCP baseline and demographic information are given in Additional file 1: Table S2. Eighteen of the PCPs interviewed were MDs, two were NPs, and one was PA. The small number of non-MDs prevented us from making comparisons between these types of PCPs.

#### PCP reactions to the reports

##### PCP understanding of and preferences about risk information

Overall, most PCPs understood the information, though two of the PCPs read the percentile as an absolute risk, for example in responding to the 75th percentile report, “in this report it does say that out of a hundred people, 75 people will get the cancer.”

PCPs, like patients, expressed a strong desire for absolute risk information. Many PCPs were “doing the calculation,” multiplying the prevalence with the odds ratio to get an estimate of absolute risk. A handful of PCPs saw the percentile as possibly useful, but many commented that it was not useful: “I don’t actually care what the 70 percent is. Because at the end of the day, I’m treating an individual.” For considering the choice of risk metric (odds ratio or some measure of absolute risk), some PCPs mentioned that there are already standard ways to think about risk for some phenotypes. Some PCPs, like some of the patients, wanted an indication of genetic attributable risk, i.e., how much of an individual’s overall risk for a disease is accounted for by the information presented.

A small number (4/21) preferred the binary report design. This was mostly because they thought it would be easier for patients to understand. The “fear of missing something” also formed the basis for one PCP’s preference for a binary report: “I think that's what keeps people up at night: I missed something.” But the majority saw large downsides to not giving a continuous value. Many PCPs had concerns for those just below the threshold, including false reassurance for those just below the threshold, and the sense that it was paternalistic not to share the continuous result with the individual.

PCPs had diverse reactions regarding whether a threshold for high risk should be provided on a report. Many would not want a threshold; these PCPs were comfortable with fitting into a gray area, particularly because patients are different: “People do have different preferences. And I think given their preferences of an opportunity to use the score, I think that's more appropriate than the more paternalistic approach of picking high or low.” Many would want a suggested cutoff. Some PCPs expressed a desire for several risk categories.

One PCP displayed genetic determinism, though, as for the patients, this was coupled with a sense that they could do something to avert it: “I need to do something, whether it’s ninety nine percentile or ninety five percentile, it doesn’t matter to me. I know that the risk is high and that this person will develop prostate cancer in the future. So I’m going to take action.”

Several PCPs emphasized the importance of the design of the report not just to convey information, but to structure the patient-PCP interaction, to help the PCP “walk them through it.” This was connected with the sense that report design can make or break the patient’s understanding: “30 years of doing this, almost every patient can explain really complicated ideas if you present the information to them correctly.” And in connection with the short shrift usually given to the design of the report: “And how do you take something as complicated and make it simple? That is very challenging. That requires UI/UX experts… that is something that in health care is bizarrely nowhere on the list.”

PCPs emphasized that report design needed to work for a diversity of patient preferences and literacy: “I think what tends to happen is that you have some patients [that] will want very, very granular detail… And then there are going to be other patients who are just .. like well, I’m at high risk.” Additionally, “You’re going to have some patients who are even challenged to understand risk and benefit discussions …. certainly you don’t want to mislead somebody with a number… you really have to give them an idea of what the number actually represents.” Some PCPs stressed the need for easily understandable patient materials, in several languages.

##### Communicating differential performance by population groups

Many PCPs were confused about the relation of the differences in performance by ancestry group with different prevalence base rates by self-reported race/ethnicity. Some wanted prevalence by population group. Many thought that the relationship between the ancestry limitation and reporting the odds ratios by ancestry group was not clear. At a deeper level, there was some confusion over whether the differences reflected our current knowledge (as indicated on the report) or true differences in disease prevalence: “If I had an Asian patient, a person of Asian ancestry in front of me, I would say that the chances are higher for developing prostate cancer because of the ancestry.”

Some PCPs were comfortable with communicating this limitation, using “take this result with a grain of salt” language. Many drew analogies to other areas of medicine where data was similarly biased, for example in risk scores for certain conditions. On the other hand, many would struggle to communicate the ancestry limitation, and were unclear about what the implications were for the patient in front of them: “The last limitation says that score has been best validated in European ancestry... So does best validated mean that it’s the highest odds? I doubt it. I suspect that it means that there’s stronger research, but I wouldn’t know how to interpret that.” One PCP came up with their own interpretation: “As best as we can tell, it's underestimating your risk [of prostate cancer] as an African-American. And so I’m going to throw in another 10 or 20 percent.”

Some PCPs brought up the subject of the population categories used on the report. Some were comfortable with the categories: “We’re in a situation in our society where, like we’re so used to classifying groups of people this way.” Some PCPs expressed concern about what to do with an individual who didn’t neatly fit into a category, though a practical response was to look at the relevant ranges and say “something in there.” Some wanted an acknowledgement that this was difficult to interpret because few people fit neatly into a category. Some thought we did not need to break down by categories if the clinical action would not change.

Most PCPs used continental ancestry categories interchangeably with racial categories, for example “I think it is interesting to know that it’s better validated in European ancestry. I think we kind of tend to know that, but. So pretty much everything is validated in Caucasians.” This was particularly true when PCPs imagined how they would explain the report to patients: “And so specifically thinking of my Latino patients and my Black patients that, you know, ‘unfortunately, these scores historically have looked at greater white population than populations that look like you.’”

#### PCP perceived utility of PRS

**Fig. 1:**
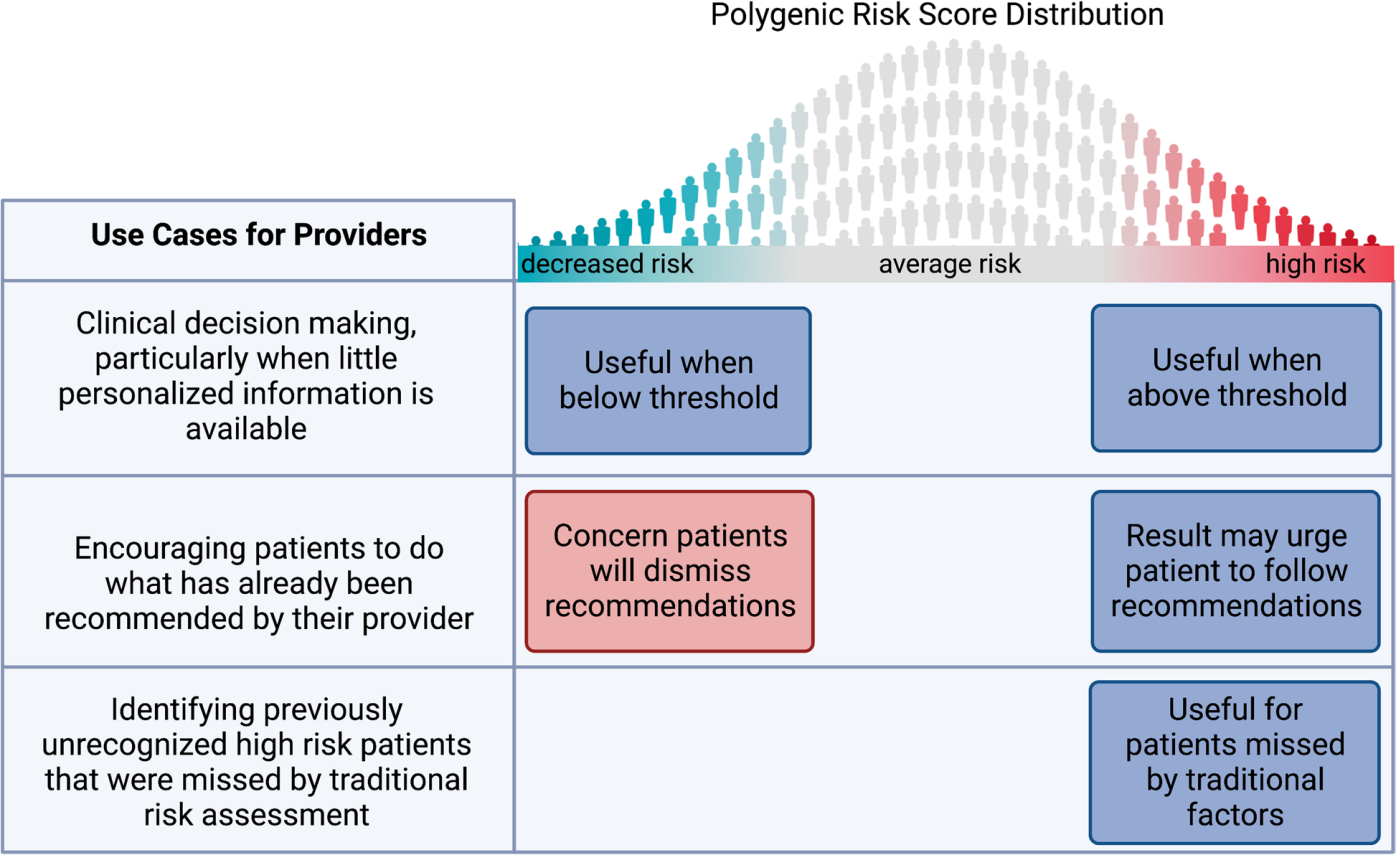
Three use cases for polygenic risk score (PRS) reports. These use cases for PRS emerged from the interviews with primary care providers (PCPs). PCPs also recognized the utility of PRS reports as an education tool, spanning these use cases. The relevance of lower than population-average-risk information varied by use case

In the examination of how PCPs would use PRS information, instead of a simple case of deciding whether or not the information was “actionable,” a more complicated picture emerged of three different use cases with very different perceived utility and engagement with the information (see Fig. 1).

##### Use Case 1: Clinical decision-making in the gray areas of patient care

PCPs saw value in personalized information informed by PRS for gray-area decision making: “Benefit, I think when there is a gray area and I feel like we can’t really tailor the recommendation as much as I want to. I think then it is definitely beneficial to have additional sort of data and scores to help guide both myself and the patient.” Some emphasized that this utility would be condition specific: “I think it depends on the condition and, you know, whether there really needs to be that kind of shared decision making and kind of risk calculation going on.” In general, PCPs were much less enthusiastic about using polygenic risk information for deciding whether or not to prescribe a medication; in this case, they would want to see proof of benefit.

For gray-area decision making, many PCPs perceived value in results below average population risk, ie. in the negative tail of the distribution. “If you had [a] report that says your patient is at low risk ...that would then probably guide a PCP like me to be more conservative in their recommendation for screening.” This was in the context of feeling that they might be “overdoing” certain tests. However, some would not use the lower risk in this way: “So I would use this basically to identify those who I might screen more regularly, but I wouldn’t use a low risk here to screen less regularly.”

PCPs used diverse heuristics for integrating a PRS into their holistic risk interpretation for the patient in front of them. Some PCPs operated with rules of thumb. Most used the odds ratio, for example, “I think that sort of one and a half increase cutoff is where it really helps to push me.” For a small handful their rule of thumb related to the percentile: “for anything more than 50 we should do a little more something.” Some were aware they did not have a good rule of thumb. A small handful of PCPs explicitly talked about the fact that PCPs were natural Bayesian integrators, “This is Bayes theorem sort of thing…. You actually have to have a fairly big increase in risk for a risk score to be useful... for most people it doesn’t make a difference because most people are either really low or really high to start with.” One PCP was aware that they should be operating in a Bayesian fashion, but was not willing to do this “naturally,” instead wanting explicit pre-test probabilities. In practice, this diversity of heuristics led to different PCP responses to the 75th percentile report, with some changing their recommendations based on this information and others refraining from doing so. Across the PCPs, the way they would use PRS information was consistent with how they would use other pieces of risk information such as family history or clinical risk factors.

##### Use Case 2: Encouraging patients to follow already recommended actions

PCPs perceived value in using PRS reports to encourage patients to do whatever they thought those patients should be doing anyway, in particular, to adopt recommended screening and a healthy lifestyle, using phrases such as “pushing” patients, providing “ammunition,” or “fuel for the conversation.” This use was noted in particular for overcoming the effects of a negative family history. “I mean many people won't do a colonoscopy. But if you’re presented with saying, ‘Hey, you're in the ninety-ninth percentile’ and you give this to them, they’re going to think very, very differently regarding it. So it could be a tool to sort of gently — or maybe not so gently — kind of push people to go get their screening updated.” Some expressed qualms about doing this, but would do it anyway: “The place where it would make a difference is if I really thought somebody should be taking a statin, for instance, or doing something with their blood sugars, it would give ammunition to do so, which is really not the right way to use these things, because it's not really doing shared decision making. It's using an argument that's unproven to get a patient to do something that you think they should do, even though they're hearing the data and don't want to do it.” For this use case, there was a concern about sharing below-population-average risk, on the basis that this might cause false reassurance.

##### Use Case 3: Identifying those at high risk who would otherwise be missed

PCPs saw value in PRS reports as a “hook” to have conversations that they might not otherwise have had. The foremost benefit of this was to detect disease early in those they might have otherwise missed: “But the benefits, I think, are huge. I think it’s going to catch people who would have been missed by traditional screening metrics. And I think that's the real benefit.” This was seen as particularly true when there is no perceived harm to screening, for example, A1C tests for diabetes.

There was also a perceived benefit to educating patients about their risk that is not achievable with other risk factors, “I think this has the potential to educate patients about themselves in a way that’s very hard in primary care…. the genetic scores indicate a really high risk independent of all these other things that we spend a lot of time thinking about, and that could be a huge actual time saver and actually be really efficient and really effective for patient education.”

##### How PRS reports could fit into PCP practice

Across these use cases, most PCPs saw using PRS reports as a natural extension of their practice. This use would fit into their existing ways of thinking about risk. Many emphasized that their practice has been trending in this direction over the last decade or so: “We all live in the risk score business now.”

Only a small handful would refer their patients to a specialist (in this case, a urologist). If asked, many said they would refer to a genetic counselor, but few brought this up before prompted. In the cases where they did bring it up, it was most often in connection with dealing with an anxious patient, and less often for help interpreting the results for a patient with many questions. PCPs frequently expressed wanting guidance on whether and when they should refer to a specialist or a genetic counselor.

Many saw the need for training and felt that with training, they would feel confident integrating this type of report into their practice: “And so it’s important that you arm those people with the right way to interpret it. So there has to be something that makes it so that I quickly come up to speed. So I understand the nuance, I understand the limitations.”

For gray-area decision-making, some PCPs were only willing to act on PRS info if there were evidence-based practice guidelines. Others wanted clarification on the relationship to professional guidelines via explicit statements on the report. For example, whether or not the relevant professional society approved or explicitly disapproved of the use of the PRS, and whether all patients were just being recommended standard guidelines.

Although most PCPs emphasized that PRS would be treated very similarly to other risk information they habitually deal with, a handful emphasized some differences. In addition to the quote above about the educational role of PRS information, one PCP described genetic information as different to other information because the underlying genetics does not change (unlike other tests), but interpretations do change more than other tests, concluding “I can say for certain that your potassium from five years ago is irrelevant to me. Where this may not be — I don't actually know in 10 years whether this will be as relevant, less relevant, more relevant.”

PCPs also expressed several concerns with the clinical implementation of PRS, which are given in Table 1. Chief amongst these were concerns about their own time, about the lack of an evidence base for the use of the scores, about potential adverse patient reactions, and about the potential implications of the differential performance by population group.

**Table 1:** Primary care provider (PCP) concerns about use of PRS information in their practice. The most cited concerns were overdiagnosis and overtreatment, lack of PCP time, and concerns over patient response

Concern	Illustrative quote
Overdiagnosis and overtreatment. (Particularly in the absence of evidence-based guidelines that establish whether the benefits outweigh the risks.)	“I think it biases patients towards doing something that is just a complete unknown. It’s potentially dangerous. People think of screening as being without danger and it’s just wrong.”
Ancestry limitation making it unclear the extent to which it would be useful for all	“You have a statement down here that these scores are best validated in people who are of European ancestry. So if I’m speaking to my African American patients, wow, I just told them that they’re disempowered . ...This is a bad tool for you, sorry. So that feels bad. And so I don’t like that idea a lot.” (Question a patient might have) “So you told me that this isn't validated in individuals of non-European ancestry. Like what? Like how come you ran this test for me and what and why do you think that the findings apply?”
Link to health disparities	“For patients we have to worry about .. how much do I have to pay for this? ... I want to have this available for all our patients, if it’s gonna be there.”
PCPs were concerned about their own:	
Lack of time for interpreting results and responding to anxiety	“This is going to take a lot of time and it’s going to take a lot of energy and it’s going to take a lot of, I think, complex, nuanced understanding of what these bell curves mean and how they impact conversations with patients.”
Insufficient understanding	“I don’t think I have the vocabulary or training to really be able to do this in an ethical or reasonable manner.”
Legal liability if someone is “missed”	“If you send me information about a patient and I don’t act on it and it’s high risk... Now they come down with the disease and somebody goes back in the medical record and says, Dr, you missed it. You're liable. And I am.”
PCPs were concerned about their patients’:	
Anxiety, in particular unnecessary anxiety	“I can definitely picture the patient that will have some amount of a meltdown over these results and sort of figuring out how to manage that is going to be important.”
False sense of reassurance	“I would worry that patients wouldn’t be able to interpret that the right way, like if they’re labeled high risk. I think that’s very easy for people to wrap their minds around. But if they’re labeled low risk by the polygenic screen, ... they might just take to heart that they're low risk, even though based upon family history or something else, they might be actually a higher risk.”
Misinterpretation of results	“I think that it is complicated and I worry that patients might not interpret it correctly.”
Insurability	“Do I decrease their chances of getting life insurance? Yes, because, you know, everything is so kind of transparent these days that even one thing in the chart can change so many things.”

Overall, almost all PCPs saw the information as useful, though a small handful did heavily circumscribe this: “To be completely frank though, not a high priority for me in terms of if I had to choose what I got more of in primary care, in terms of resources. I just think there are a lot of other things before this that we really need.”

PCP responses were relevant to many reporting choices. We combine these with insights from the patient interviews in Points to Consider in the clinical reporting of PRS (see the “Discussion” section and Table 2).

**Table 2:** Recommendations in designing PRS reports. Integrating perspectives from both patients and PCPs, we offer the following points to consider for PRS clinical report design. Many of these recapitulate best practices from the risk communication literature; some are PRS-specific. See Discussion for the highlighting of certain points, in particular for a continuous (versus binary) sharing of risk information, the clear advantage of sharing absolute risk information, and the handling of differential performance of PRS by population group

Need/concern identified	Recommendation
Based on points emerging from both patient and PCP interviews	
Both patients and PCPs were frustrated in the absence of granular risk information.	Give either a continuous estimate or category of risk, e.g., amongst a five-category framework.
Both patients and PCPs expressed a desire for absolute risk information. Absent absolute risk, PCPs will “do the calculation,” multiplying odds ratio with disease prevalence to come up with an approximate absolute risk.	Give absolute risk if possible. While an absolute risk incorporating all known risk factors is preferable, just incorporating age and sex and appropriate base rates is an improvement over giving relative risk.
Many patients and some PCPs interpreted the percentile as an absolute risk.	Be wary of potential misinterpretations of percentile.
Both patients and PCPs expressed the desire to know the amount of overall risk captured by all genetic factors, and the amount of risk captured by the PRS.	Give the attributable risk for the PRS in question, and all genetic factors.
Both patients and PCPs wanted to know how PRS information related to family history information, e.g. is it independent, or additive? The desire for this information holds even if an integrated score is used, to aid in overall understanding of the significance of PRS information.	Explain the relationship to other risk factors, in particular family history.
Population categories described in terms of continental ancestry categories are likely to be interpreted as racial groups by both patients and PCPs; Patients and PCPs were both uncertain about how the results would apply to someone who didn’t fit neatly into one of the groups; PCPs wanted to understand why the numbers were different for the different population groups; PCPs had confusions about how these differences related to different disease prevalence rates in different racial groups.	Don’t use population categories if the clinical implications of the information are the same for each category. If using population categories, (a) make a statement that not everyone falls into one of these categories, and what should be done in this case, (b) clarify why these numbers are different, and c) clarify how this information relates to different disease prevalences.
Both patients and PCPs had questions including whether they should have less confidence outside of European ancestry individuals, and how likely the PRS is to be underestimating risk in certain populations	Any statement about limitations of the information in different ancestry groups should make clear what the implications are for patients.
Both PCPs and patients wanted to know what the implications of PRS information was for family members, including under what conditions, if any, family members should be tested.	Explain implications for family members.
Many patients responded well to a graphical representation of the data, and PCPs found a graph to be a useful talking point.	Use graphical representations.
Patients anticipated feelings of anxiety and concern for receiving high risk information, and also of feelings of being “off the hook” if they were not identified as at high risk. PCPs were concerned that their patients would have these same reactions.	Talk to the patient about potential emotional responses to a report during pre-test counseling and informed consent.
Many patients mentioned the emotional valence associated with the color red, it can create unnecessary patient anxiety by placing a judgment on a piece of information. It can also prompt action. Some PCPs mentioned that red can also prompt possible PCP overreaction.	Red should be used carefully or avoided entirely.
Based on points emerging from patient interviews	
Some patients did not understand the meaning of any of the numbers shared. Others understood all the quantitative information and wanted more. Many just engaged with one component of the report.	Plan for diversity in patient ability to understand, and desire for, quantitative information. In particular, plan for selective engagement with the report; it should not be possible to come away with the “wrong” view if they just looked at one bit.
Based on points emerging from PCP interviews	
Some PCPs utilize rules of thumb; others want to know pre-test probabilities; others will only act on professional guidelines.	Plan for diversity in how PCPs think about individual risk factors.
PCPs view the report as more than a way to convey risk information. The report can act as a tool to structure the flow of the conversation.	Design report with the way it will structure the conversation between the patient and PCP in mind.
Many PCPs wondered why monogenic information was not included, and wouldn’t have been able to answer patient questions on this point.	Ideally, monogenic risk should be incorporated into an aggregate genetic risk score. If this is not possible, the report should explain why monogenic risk information is not incorporated into the PRS.
Some PCPs stressed that after the conversation with the PCP, the patient should have materials that they can understand.	Ensure availability of accessible, easy-to-understand patient materials, in multiple languages.
Some PCPs expressed a preference for materials to help them understand what a PRS is and provide succinct explanations suitable for the short time period allotted for clinical visits.	Provide materials to help physicians get comfortable with their “spiel”; these points should help guide the flow of conversation.
PCPs wanted to know answers to such questions as: Do professional societies approve of the use of this type of information? Do they actively disapprove? Are listed recommended actions those that guidelines already recommend for someone of average risk?	Given that there are not currently published guidelines for healthcare based on PRS, clarify relationship of recommended actions to existing guidelines (e.g., if recommendations given follow standard guidelines for those of average risk, state this)

## Discussion

Overall, high numeracy patients understood the information presented to them and often had a desire for more information. In contrast, other patients struggled to understand any of the numerical information. Despite patient misunderstanding of details of the report, most patients understood the report at a high level — i.e. the report indicated high risk and there were things that could be done to lower the overall risk. Most of the questions the patients would have for their PCPs were “what can I do?”, rather than “what does this mean for me?”. Given how new PRS are and the limitations for individuals of different ancestries, the fact that patients are taking their value as a given is notable. Patients often displayed both a feeling that the information was deterministic, using language like “definitely going to get the disease” and the belief that there was something they could do to lower their risk. Patients typically engaged selectively with the report, not looking at all information presented, and not engaging with the limitations section. A substantial minority of patients would anticipate a negative emotional reaction to receiving high risk information. The vast majority of the patients preferred the continuous report design, both because they would want to know their exact risk and because they appreciated the graphic that the continuous report design included. When explicitly asked, most would want a report even if inaccurate for their ancestry. Our results suggest that PCPs should be sure to talk through the meaning of the results with patients and ensure patient understanding.

The PCPs had some confusion about the information displayed on the clinical reports, and many questions about their accurate interpretation. They shared concerns about the ancestry limitation. Like the patients, the PCPs overwhelmingly preferred the continuous report design. Three main use cases for PRS in primary care emerged. Across all these uses, PCPs mostly considered that their use of PRS would be continuous with their current practice, resembling the ways they already think about other risk factors for common conditions. The delineation of these three use cases for PRS that emerged from the PCP interviews — clinical decision-making in the gray areas of patient care, encouraging patients to follow already recommended actions, and identifying those at high risk who would otherwise be missed — can help sharpen ongoing research into the clinical impact of PRS, particularly when it comes to the design of interventions.

**Fig. 2:**
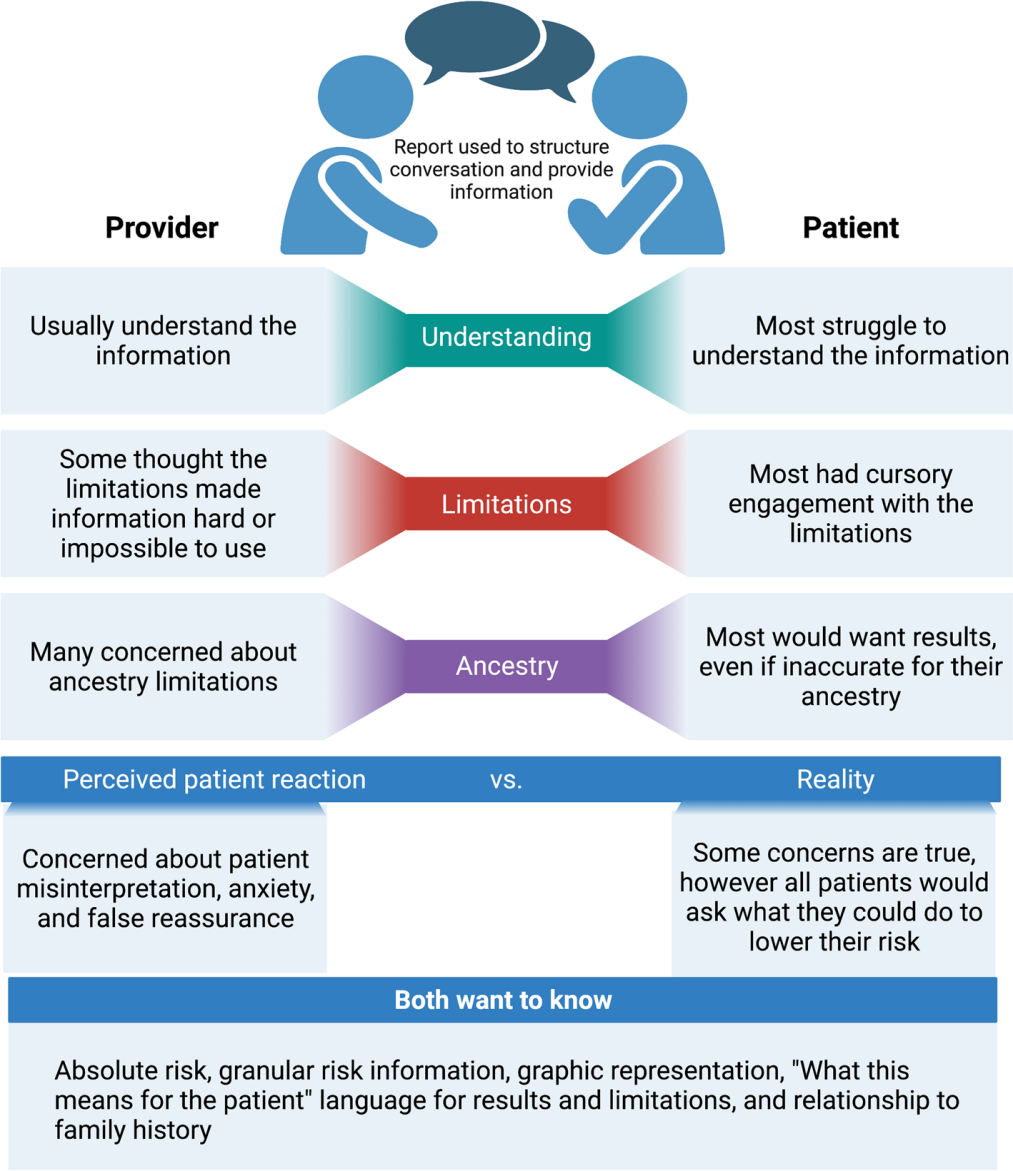
Patient and primary care provider (PCP) interaction around polygenic risk score (PRS) reports. Synthesizing the reactions we observed from both patients and PCPs, we observe several points of agreement and highlight the multiple ways in which the PCP has a role within a conversation about risk

There are clear points of agreement between PCPs and patients and some clear ways in which the PCP has a role within a conversation about risk, for example, in aiding accurate interpretation, contextualizing the report within limitations, and recommending actions. In Fig. 2, we illustrate how these insights from our patient and PCP interviews interweave.

A key element of any integration of PRS into clinical care is the design of the clinical report, which can have a large impact on the ethical clinical implementation of PRS. There are several important decisions to consider in the PRS report design. These include: whether the risk level is contextualized as a percentile, a measure of relative effect, or an absolute risk [35]; whether the risk is presented in a binary or continuous fashion; how and whether the differential performance of PRS by population group is communicated.

Many of our results help establish that prior findings from the risk communication literature generalize to PRS. We found evidence that the percentile is often misinterpreted — by patients as well as a small number of PCPs — as an absolute risk. Measures of relative effects, such as odds ratios, are frequently overinterpreted [19]. For this reason, measures of absolute risk are typically considered a preferred communication tool. We observed a strong preference amongst both patients and PCPs for absolute risk information. In the absence of this, many PCPs were attempting to estimate absolute risk based on the prevalence and odds ratio. This suggests that reporting absolute risk should be a priority for the clinical use of PRS. To date, absolute risk models are not part of standard care for the majority of conditions, with breast cancer and coronary heart disease being notable exceptions [36, 37]. But while the gold standard for use of an absolute risk model includes incorporation of multiple risk factors from multiple longitudinal cohort studies, evidence of the population-based prevalence of risk factors, and evidence-based guidelines tied to thresholds of risk, our results suggest that simple absolute risk models, perhaps just including age and sex, may be more appropriate than not reporting absolute risk at all. There is already a tool to convert a PRS into absolute risk solely via the incorporation of disease prevalence [38].

Our patient and PCP interviews also revealed a very strong preference for reporting PRS information in a continuous rather than binary way. This strong desire by the patient participants for exact risk information should be considered alongside the low levels of understanding of the numerical risk information that we observed. Some PCPs highlighted the inappropriateness of providing a threshold alongside a continuous measure of risk for cases of shared-decision making.

The negative emotional reactions that the patient participants anticipated if they were to receive actual (as supposed to mock) high risk results should be considered alongside empirical work on the return of actual genetic results. Here, the consistent pattern has been that patients do not have sustained negative psychological reactions to receiving unfavorable genetic information, with the exception of Huntington’s disease risk [39–41]. There is also emerging evidence that this lack of negative psychological reaction may extend to PRS [10, 42] though the evidence for this is not uniform [17].

Considering the reactions of patients and PCPs to mock reports, Table 2 gives some Points to Consider in the design of clinical PRS reports. We stress that the design of a PRS report needs to reflect the context in which it will be read, for example, the needs of the patient population, and whether it will be disclosed by a provider, and if so, what education they can be assumed to have had. Many of these Points to Consider reflect conclusions established across risk communication contexts; others are specific to PRS. Some of these suggest additional research is needed on the PRS development side, for example how a PRS relates to family history and the implications for family members. An implication of our results is that PRS research should expand the investigation to ensure this information is available to downstream clinical users.

A particularly challenging decision facing those implementing clinical PRS is how to handle the differential predictive performance of PRS by population group. It is not clear what is driving these differences in predictive performance. While these differences may simply reflect the fact that we have more genomic and health history data in some populations, they may also represent divergent population histories (typically framed as reflecting differences in patterns of linkage disequilibrium) as well as structural determinants of health [43, 44]. This issue of differential performance by population group resulted in one early PRS clinical report, from Ambry genetics, being pulled from the market [16], and motivated a major re-release of another prominent clinical PRS, from Myriad Genetics [27]. Complicating clinical reporting of differential performance by population group is the fact that researchers validate PRS using data sets that label individuals in different ways — by self-reported race and/or ethnicity, inferred race and/or ethnicity, genetically inferred categories representing genetic similarity (typically continental ancestry categories), a mixture of the above, and others in not clearly specified ways [8, 45]. Conflating race/ethnicity and continental ancestry categories is common in interpreting genetic studies [46]. If differences in predictive performance by population group are reported, healthcare providers and/or patients might inaccurately interpret them as reflecting meaningful between-group biological differences. Our results highlight the dangers of reporting results separately by population group. In particular, many individuals do not neatly fit into any of the groups, and some individuals likely fit into more than one group. Additionally, the predictive performance of PRS is not the only thing that can systematically vary between population groups, and it is not clear how this variation intersects with, for example, the different prevalence of disease in different populations. Recent attention to the misuse of race as a variable in clinical support decisions should make us wary of integrating population group descriptors into clinical reporting [47]. While the use of continental ancestry categories may seem like a more objective grounding for reporting these performance differences, their use is also highly problematic because they perpetuate the incorrect idea that humans come in a small variety of biological types [48, 49]. Our recommendations (as given in Table 2) include that differences in performance of PRS by population group should only be given if these differences would lead to different clinical recommendations in these different groups. Our finding that patients desired results even if inaccurate for their ancestry is in keeping with a similar perspective shared by African Americans in conjunction with Alzheimer’s disease risk results [50].

Our results directly informed some aspects of the report design and education for PRS reporting in the eMERGE IV network. The decision to report results in a binary rather than continuous fashion was taken early in eMERGE IV before our results were ready, though our results did inform the use of a similar graphic to that used on our continuous report design. The finding that patients wanted their scores even if inexact for their own ancestry group informed the decision to include PRS scores validated in two or three ancestry groups and not just those validated in the four ancestry groups. Our observations about genetic determinism informed how risk is discussed in the aggregated risk report. The conflation between continental ancestry categories and racial categories that we observed helped inform the network’s choice of how to describe these populations. The points of confusion we observed in both sets of interviews informed how the numerical information was explained on the reports and education materials.

One limitation of our study is that those who volunteered for our study — both patients and PCPs — may have more interest and/or a better understanding of genetics than the general population, though we note that we had a wide range of both health and genetic literacy amongst our patient participants. A limitation of our patient interviews is that all participants received education about PRS prior to being asked to interpret the reports. This amount of education is more than patients would receive in practice, so our participants may have been better able to interpret the clinical reports than a general population. Limitations of our PCP interviews include that all practice at clinics affiliated with a major urban academic medical center. While many of these practices were from community health centers serving diverse communities, we are missing perceptions from those who practice in rural communities, and in other parts of the country which may have meaningfully different reactions to PRS. The fact that our mock reports for the PCP interviews were designed for prostate cancer may also be perceived as a limitation, although the discussions in the interviews ranged widely over other conditions. Additionally, some misunderstandings and misinterpretations could reflect problems in the way the questions were phrased or with the ways we chose to present the visual data: we made many decisions about how to display this information and other decisions could have led to improved comprehension (identifying improved ways to present the information was a major motivation for this work).

## Conclusions

In our interviews with patients, we found that most patients were not able to accurately interpret mock results for disease x based on the PRS report. However, all patient interviewees nonetheless got a key take home message that they were at increased risk and that there were actions they could take to lower their overall risk.

Our interviews with PCPs indicated that they see utility in incorporating PRS in their conversations with patients. Beyond the limitations on their time, the key barrier PCPs see is the lack of evidence-based guidelines for their use. The single most useful advance for the use of PRS in the clinic would thus be clinical outcomes data linked to professional guidelines. Implementation studies such as eMERGE IV may provide such data in the future. PCPs perceived value across three use cases, suggesting that clinical use of PRS should not be thought about monolithically, but also suggesting that there may be some considerations that are relevant across conditions. PCPs emphasized that the use of PRS would fit into their existing ways of thinking about risk. This is a fruitful avenue for further research on a condition-by-condition basis.

Considered jointly, our patient and PCP interviews revealed that how this risk information is conveyed will be a crucial determinant of the overall impact of PRS. There are many components of risk communication, including both patient and PCP education and numeracy levels. Clinical report design is an important piece of this risk communication, and careful design that incorporates stakeholder perspectives could help realize the perceived value and minimize some of the concerns associated with the use of PRS. The Points to Consider we share here — which recapitulate the relevance of prior findings from the risk communication literature as well as highlighting some PRS-specific considerations — offer a first step towards the development of guidelines for the clinical reporting of PRS. In particular, we emphasize opting for a continuous (rather than binary) display of information, the use of absolute risk as opposed to the percentile rank or a measure of relative risk (even if the absolute risk model is a simple one and not in widespread use), and tying consideration of communicating differential performance by population group to whether or not clinical recommendations would vary by these groups.

There are many avenues for further investigation to enable the field to identify and establish best practices for reporting PRS. Our study suggests the need to investigate effective ways to dispel genetic determinism. Because our PCP interviews highlighted that adoption of PRS will depend closely on the nature of existing clinical guidelines for risk management, how report design should vary depending on this availability should also be studied. Finally, our study leaves many open questions about how best to communicate the differential performance of PRS by population group, including the choice of population descriptors, the choice of populations, whether these differences should be communicated, and if so how they should be explained, and indeed the conditions under which a PRS should be reported at all if these differences are very large

## Supplementary Information

**Additional file 1: Tables S1** and **S2.** Further information on patient survey items; Patient interview guide, with mock report designs; Primary Care Physician interview guide, with mock report designs.

## Data Availability

The anonymized transcripts collected and analyzed during the current study are available from the corresponding author on reasonable request (these are not being made publicly accessible per the informed consent document approved by the IRB).
