# Supplementary material for: Patient and provider perspectives on polygenic risk scores: implications for clinical reporting and utilization
Source: Genome Med. 2022 Oct 7;14:114. doi: 10.1186/s13073-022-01117-8 (PMC9540716; doi:10.1186/s13073-022-01117-8)
Supplement: Supplementary file 1 — Additional file 1: Tables S1 and S2. Further information on patient survey items; Patient interview guide, with mock report designs; Primary Care Physician interview guide, with mock report designs. [file 13073_2022_1117_MOESM1_ESM.pdf]

Additional File 1 for “Patient and provider perspectives on polygenic risk scores: Implications for clinical reporting and utilization”

Contents:

- Tables S1 and S2
- Further information on patient survey items
- Patient interview guide, with mock report designs
- Primary Care Provider interview guide, with mock report designs

Table S1: Patient demographics and baseline information (N =25)

**Self-identified race/ethnicity**	Non-hispanic black	5
	Non-hispanic white	5
	Non-hispanic Asian	5
	Hispanic white	10
**Age**	20-29	4
	30-39	6
	40-49	4
	50-59	7
	60-69	1
	70-79	3
**Gender**	Male	8
	Female	17
**Genetic literacy**	Low (<9)	5
	Average (10-17)	14
	High (18+)	6
**Numeracy**	Low	3
	Average	13
	High	9
**Health literacy**	Inadequate	9
	Adequate	16

Table S2: Primary Care Provider (PCP) demographics and baseline information (N=21)

**Primary care provider (PCP) type**	MD	18
	PA	2
	NP	1
**Gender**	Male	11
	Female	10
**Self-identified race**	Black	2
	East/Southeast Asian	5
	White	14
**Years of practice**	0-3	3
	4-9	8
	10-19	2
	20-29	5
	30-39	1
	40+	2
**Half day practice sessions per week**	1-2	3
	3-4	6
	5-7	9
	8+	3
**% patients from minority groups**	10-20%	5
	21-40%	7
	41-50%	3
	>50%	6
**Had heard of PRS**	Yes	12
	No	9

Further information on the additional instruments used in patient interviews

For the University of North Carolina Genomic Knowledge Scale (1), the mean in the published validation was 14-15, A score of 8 is one SD below, and we coded scores of 1-8 as low genetic literacy. A score of above 17 is one standard deviation above, and we scored scores of 18-19 as high genetic literacy. Scores of 9-17 were coded as medium.

Validation of the Subjective Numeracy Scale (2) found a median SNS composite rating of 4.2 on a scale ranging from 1 to 6, with a 25th percentile of 3.2 and a 75th percentile of 4.8 (3). We coded below 3.2 as low numeracy, above 4.8 as high numeracy, and in between these values as medium numeracy.

For the Short Test of Functional Health Literacy in Adults (4), the authors state that "Any response that is 3 or greater on any question indicates inadequate health literacy," and we followed this.

1. Langer MM, Roche MI, Brewer NT, Berg JS, Khan CM, Leos C, et al. Development and Validation of a Genomic Knowledge Scale to Advance Informed Decision-Making Research in Genomic Sequencing. MDM Policy Pract. 2017 Jan 1;2(1):2381468317692582.
2. Fagerlin A, Zikmund-Fisher BJ, Ubel PA, Jankovic A, Derry HA, Smith DM. Measuring Numeracy without a Math Test: Development of the Subjective Numeracy Scale. Med Decis Making. 2007 Sep 1;27(5):672–80.
3. Zikmund-Fisher BJ, Smith DM, Ubel PA, Fagerlin A. Validation of the Subjective Numeracy Scale: Effects of Low Numeracy on Comprehension of Risk Communications and Utility Elicitations. Med Decis Making. 2007 Sep 1;27(5):663–71.
4. Chew LD, Bradley KA, Boyko EJ. Brief questions to identify patients with inadequate health literacy. Fam Med. 2004 Sep;36(8):588–94.

eMERGE IV Biobank Participant Interview Guide

Hello, Mr./Ms. ________. Thank you for agreeing to take part in our interview study. My name is _____ and I am a research coordinator from Mass General Brigham Personalized Medicine. How are you today?

As a reminder, we are conducting a research study. The purpose of this interview is to hear your thoughts about the design of polygenic risk score reports for the research study. There are no right or wrong answers to these questions. This interview will be audio recorded and then transcribed and analyzed. All identifying information will be removed and all of the analyzed data will remain anonymous. Is it okay if I begin the recording now?

This interview will last about 45 minutes. For participating in this interview, we will send you a $25 check as a thank you for your time. Your participation in this interview is completely voluntary and if at any point you wish to stop the interview, you are welcome to do so. Additionally, if there is a question you would rather not answer, just let me know. Do you have any questions before we get started?

Experience with genetics
Interviewer: Can you tell me about any experiences you have with genetics?

- **Prompt:** If a participant is unsure, ask if they have encountered genetics while learning in school, a personal or family member/friend diagnosed with a genetic condition, your own curiosity, work, Media/TV, etc.

Besides genetics, what other factors do you think would affect your chance to develop a health condition like heart disease?

Besides genetics, what other factors do you think would affect your chance to develop a health condition like cancer?

Regardless of participant response, provide an overview of genetics: Great, now I am just going to provide you with a brief overview of genetics that will help frame our discussion about polygenic risk scores. Our genetic information is made up of DNA which is inherited from our parents. DNA is the instruction book for our body which helps to determine how our bodies grow, develop, and function everyday.

Overview of PRS
Next I will take us to a website so we can walk through the definition of a polygenic risk score.
Interviewer to follow link below and share screen with the patient and scroll through the graphics while reading the text on the left side (If patient requests, they can read on their own and you can send the link over zoom. You can stop after this sentence

> *"Even though you can't change your polygenic score, following a healthy lifestyle and working with your health care team to treat any clinical risk factors can significantly decrease your risk."*

http://polygenicscores.org/explained/

Interviewer: In your own words, how would you describe what a polygenic risk score is? (*make sure to stop sharing screen before this question so the respondent is not seeing any language on the screen)*

Continuous vs. Dichotomous

Interviewer: Now I will show you one example of a Polygenic Risk Score report. A volunteer named Louis provided a sample of his blood for a research study and we performed genetic testing to create this PRS report. His polygenic risk for Disease X is shown below. *(Interviewer shares screen of report).* Please review and let me know when you are ready for my questions.

Name: **Martinez, Louis**	MRN: **1234567**	
DOB: **01/01/1980**	Referring Facility: **BWH**	Specimen: **Blood, peripheral**
Sex: **Male**	Referring Physician: **Dr. Elizabeth Karlson**	

POLYGENIC DISEASE RISK

Polygenic risk describes the chance of developing certain health conditions based on a large number of genetic variants across the genome. This test assessed the risk for developing the following condition: Disease X

This test identified high polygenic risk for Disease X

Disease X high polygenic risk

Disease	This patient's result	General Disease Rate
X	High polygenic risk	1 in 10 people will develop Disease X in their lifetime

Based on analysis of your DNA sample, you have a high polygenic risk to develop disease X at some point in your life. Out of every 100 people, your polygenic score is higher than at least 98 people, or in other words, your polygenic risk score is in the top 2%. Polygenic risk scores in the top 2% are associated with greater than a 3-fold risk of developing disease X, according to existing data. These results do not indicate that you have disease X now or will definitely develop disease X in the future. There are still steps you and your healthcare team can take to prevent development of the disease or diagnose and treat it early.

LIMITATIONS

- This polygenic risk score does not take into account non-genetic factors such as lifestyle habits and history of other diseases, which could affect your risk.
- These results should be viewed in the context of your medical care, family history, and racial/ethnic background.
- Polygenic risk scores are looking for common genetic variants that can increase risk when added together. For that reason, this polygenic risk score does not look for single rare genetic variants in genes associated with disease X.
- The majority of existing data used to calculate polygenic risk scores are from individuals of European ancestry.

- How would you interpret this report?
  - Prompt: What do you think are the main takeaway points for Louis?
  - What does a 3-fold risk mean to you?
- Are there any immediate questions you have while looking at this report? (If patient question includes one of our questions below, ask them to try to answer themselves)
- When looking at this report, what does high polygenic risk mean to you?

    - Prompt: How likely do you think Louis is to develop Disease X in his lifetime?
    - Prompt: How much more likely would Louis be to develop this condition than the general population?
- If you put yourself in Louis' place, what questions would you have for your health care provider about your risk?
- Do you have any general comments or suggestions about this report?

Limitations:

- What is your understanding of the limitations section?
    - Prompt: What is your understanding of the last bullet in the limitations section?
- Are there any immediate questions you have while looking at the limitations section?
- How could we explain this section better?
- If you were asked if you would like to receive a polygenic risk score result, would you still like to receive this information if the results may be inexact for your ancestry?
    - What does inexact mean to you?

Interviewer: Now I will show you another report for Louis. To note, the limitations section will be identical for all future reports. Please review and let me know when you are ready for my questions.

Name: **Martinez, Louis**	MRN: **1234567**	
DOB: **01/01/1980**	Referring Facility: **BWH**	Specimen: **Blood, peripheral**
Sex: **Male**	Referring Physician: **Dr. Elizabeth Karlson**	

POLYGENIC DISEASE RISK

Polygenic risk describes the chance of developing certain health conditions based on a large number of genetic variants across the genome. This test assessed the risk for developing the following condition: Disease X

This test did not identify high polygenic risk for Disease X.

Disease X — Not high polygenic risk

Disease	This patient's result	General Disease Rate
X	Not high polygenic risk	1 in 10 people will develop Disease X in their lifetime

Based on analysis of your DNA sample, you do not have a high polygenic risk of developing X disease at some point in your life. For disease X, polygenic risk scores are considered high risk if they are in the top 2%, or In other words, out of every 100 people, the polygenic score would need to be higher than at least 98 people. Polygenic risk scores in the top 2% are associated with greater than a 3-fold risk of developing disease X, according to existing data. These results do not indicate that you have disease X now or will definitely develop disease X in the future. There are still steps you and your healthcare team can take to prevent development of the disease or diagnose and treat it early.

LIMITATIONS

- This polygenic risk score does not take into account non-genetic factors such as lifestyle habits and history of other diseases, which could affect your risk.
- These results should be viewed in the context of your medical care, family history, and racial/ethnic background.
- Polygenic risk scores are looking for common genetic variants that can increase risk when added together. For that reason, this polygenic risk score does not look for single rare genetic variants in genes associated with disease X.
- The majority of existing data used to calculate polygenic risk scores are from individuals of European ancestry.

- How would you interpret this report?
    - Prompt: What do you think are the main takeaway points for Louis?
- Are there any immediate questions you have while looking at this report? (If patient question includes one of our questions below, ask them to try to answer themselves)
- When looking at this report, what does "not high" polygenic risk mean to you?
    - Prompt: How likely do you think Louis is to develop Disease X in his lifetime?
    - Prompt: How likely is Louis to develop this condition compared to the general population?
- If you put yourself in Louis' place, what questions would you have for your health care provider about your risk?

Okay, now we will move onto a different representation of Polygenic Risk Score and I will ask similar questions. Please review the report and let me know when you are ready to discuss.

Name: **Martinez, Louis** MRN: **1234567**
DOB: **01/01/1980** Referring Facility: **BWH** Specimen: **Blood, peripheral**
Sex: **Male** Referring Physician: **Dr. Elizabeth Karlson**

POLYGENIC DISEASE RISK

Polygenic risk describes the chance of developing certain health conditions based on a large number of genetic variants across the genome. This test assessed the polygenic risk for developing the following condition: Disease X

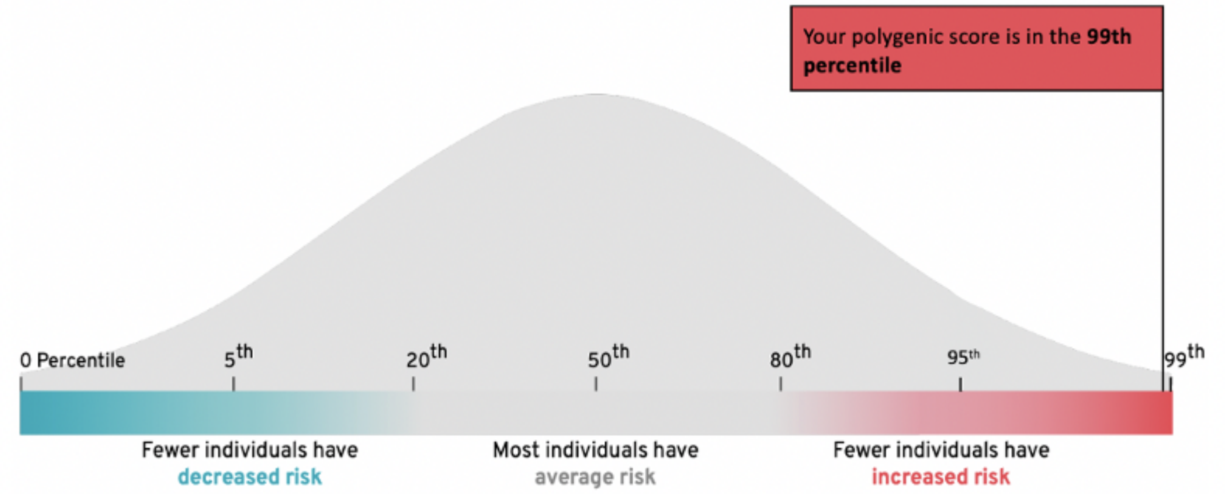

<table>
<tr><th>Disease</th><th>This patient's result</th><th>General Disease Rate</th></tr>
<tr><td>X</td><td>99th percentile</td><td>1 in 10 people will develop Disease X in their lifetime</td></tr>
<tr><td colspan="3">Based on analysis of your DNA sample, your polygenic score is in the 99th percentile. This does not mean that you have a 99% chance of developing Disease X. It means that out of every 100 people, your polygenic score is higher than 99 people, and the same or lower than 1. A polygenic risk score at the 99th percentile is associated with a 3.4-fold risk of developing disease X, according to existing data. These results do not indicate that you have disease X now or will definitely develop disease X in the future. There are still steps you and your healthcare team can take to prevent development of the disease or diagnose and treat it early.</td></tr>
<tr><td colspan="3">LIMITATIONS
• This polygenic risk score does not take into account non-genetic factors such as lifestyle habits and history of other diseases, which could affect your risk.
• These results should be viewed in the context of your medical care, family history, and racial/ethnic background.
• Polygenic risk scores are looking for common genetic variants that can increase risk when added together. For that reason, this polygenic risk score does not look for single rare genetic variants in genes associated with disease X.
• The majority of existing data used to calculate polygenic risk scores are from individuals of European ancestry.</td></tr>
</table>

- How would you interpret this report?
  - Prompt: What do you think are the main takeaway points for Louis?
- Are there any immediate questions you have while looking at this report? (If patient question includes one of our questions below, ask them to try to answer themselves)
- When looking at this report, how do you interpret the risk for Louis?
  - Prompt: How likely do you think Louis is to develop Disease X in his lifetime?
  - Prompt: How likely is Louis to develop this condition compared to the general population?

- If you put yourself in Louis' place, what questions would you have for your health care provider about your risk?
- Do you have any general comments or suggestions about this report?

Now I will show you one more report for Louis. Please review and let me know when you are ready for my questions.

Name: **Martinez, Louis**	MRN: **1234567**	
DOB: **01/01/1980**	Referring Facility: **BWH**	Specimen: **Blood, peripheral**
Sex: **Male**	Referring Physician: **Dr. Elizabeth Karlson**	

POLYGENIC DISEASE RISK

Polygenic risk describes the chance of developing certain health conditions based on a large number of genetic variants across the genome. This test assessed the polygenic risk for developing the following condition: Disease X.

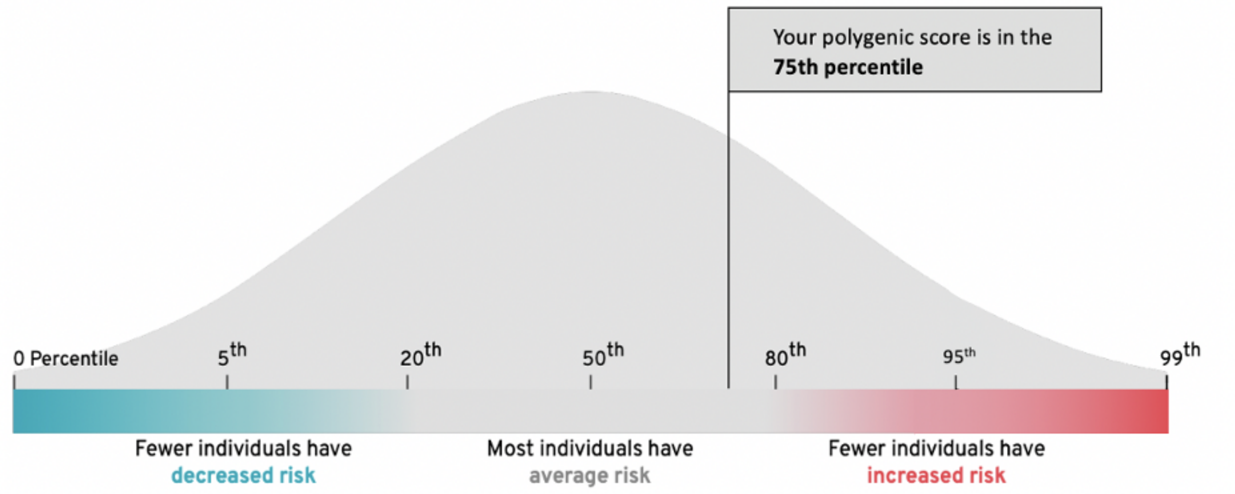

Disease	This patient's result	General Disease Rate
X	75th percentile	1 in 10 people will develop Disease X in their lifetime

Based on analysis of your DNA sample, your polygenic score is in the 75th percentile. This does not mean that you have a 75% chance of developing Disease X. It means that out of every 100 people, your polygenic score is higher than 75 people, and the same or lower than 25. A polygenic risk score at the 75th percentile is associated with a 1.4-fold risk of developing disease X according to existing data. These results do not indicate that you have disease X now or will definitely disease X in the future. There are still steps you and your healthcare team can take to prevent development of the disease or diagnose and treat it early.

LIMITATIONS

- This polygenic risk score does not take into account non-genetic factors such as lifestyle habits and history of other diseases, which could affect your risk.
- These results should be viewed in the context of your medical care, family history, and racial/ethnic background.
- Polygenic risk scores are looking for common genetic variants that can increase risk when added together. For that reason, this polygenic risk score does not look for single rare genetic variants in genes associated with disease X.
- The majority of existing data used to calculate polygenic risk scores are from individuals of European ancestry.

- How would you interpret this report?
  - Prompt: What do you think are the main takeaway points for Louis?
  - Prompt: What does a 1.3-fold risk mean to you?

- Are there any immediate questions you have while looking at this report? (If patient question includes one of our questions below, ask them to try to answer themselves)
- When looking at this report, how do you interpret the risk for Louis?
    - Prompt: How likely do you think Louis is to develop Disease X in his lifetime?
    - Prompt: How likely is Louis to develop this condition compared to the general population?
- If you put yourself in Louis' place, what questions would you have for your health care provider about your risk?

- Of the two report designs I've shown you, which report picture do you prefer? Why?
    - Prompt: Would you like to know your exact polygenic risk?
        - Why or why not?
    - If patient chooses continuous:
        - Did you prefer the continuous report because you would rather have a picture to look at? Or because you prefer knowing your exact polygenic risk? Or both?

Now we will move on to the last section. We would like to see how participants respond to seeing polygenic risk scores for several conditions. Please review and let me know when you are ready to discuss. Please note that the limitations section is not provided, but is identical to the previous limitations.

POLYGENIC DISEASE RISK

Polygenic risk describes the chance of developing certain health conditions based on a large number of genetic variants across the genome. This test assessed the risk for developing the following conditions: Disease W, Disease X, Disease Y, Disease Z.

This test identified a high polygenic risk for Disease Z. It did **NOT** indicate increased polygenic risk for the remaining conditions.

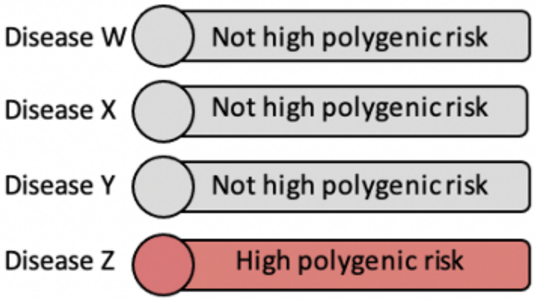

Diseases WITH an increased polygenic risk

<table>
<tr><th>Disease</th><th>This patient's result</th><th>General disease rate</th></tr>
<tr><td>Disease Z</td><td>High polygenic risk</td><td>1 in 15 people will develop Disease Z in their lifetime</td></tr>
<tr><td colspan="3">Based on analysis of your DNA sample, you have a high polygenic risk to develop disease Z at some point in your life. Out of every 100 people, your polygenic score is higher than at least 98 people, or in other words, your polygenic risk score is in the top 2%. Polygenic risk scores in the top 2% are associated with greater than a 3-fold risk of developing disease Z, according to existing data. These results do not indicate that you have disease Z now or will definitely develop disease Z in the future. There are still steps you and your healthcare team can take to prevent development of the disease or diagnose and treat it early.</td></tr>
</table>

Diseases WITHOUT an increased polygenic risk

<table>
<tr><th>Disease</th><th>This patient's result</th><th>General disease rate</th></tr>
<tr><td>Disease W</td><td>Not high polygenic risk</td><td>1 in 20 people will develop Disease W in their lifetime</td></tr>
<tr><td colspan="3">Based on analysis of your DNA sample, you do not have a high polygenic risk of developing disease W at some point in your life. For disease W, polygenic risk scores are considered high risk if they are in the top 1%, or in other words, out of every 100 people, the polygenic score would need to be higher than at least 99 people. Polygenic risk scores in the top 1% are associated with greater than a 2.8-fold risk of developing disease W, according to existing data. These results do not indicate that you have disease W now or will definitely develop disease W in the future. There are still steps you and your healthcare team can take to prevent development of the disease or diagnose and treat it early.</td></tr>
</table>

<table>
<tr><th>Disease</th><th>This patient's result</th><th>General disease rate</th></tr>
<tr><td>Disease X</td><td>Not high polygenic risk</td><td>1 in 12 people will develop Disease X in their lifetime</td></tr>
<tr><td colspan="3">Based on analysis of your DNA sample, you do not have a high polygenic risk of developing disease X at some point in your life. For disease X, polygenic risk scores are considered high risk if they are in the top 4%, or in other words, out of every 100 people, the polygenic score would need to be higher than at least 96 people. Polygenic risk scores in the top 4% are associated with greater than a 2.5-fold risk of developing disease X, according to existing data. These results do not indicate that you have disease X now or will definitely develop disease X in the future. There are still steps you and your healthcare team can take to prevent development of the disease or diagnose and treat it early.</td></tr>
</table>

<table>
<tr><th>Disease</th><th>This patient's result</th><th>General disease rate</th></tr>
<tr><td>Disease Y</td><td>Not high polygenic risk</td><td>1 in 30 people will develop Disease Y in their lifetime</td></tr>
<tr><td colspan="3">Based on analysis of your DNA sample, you do not have a high polygenic risk of developing disease Y at some point in your life. For disease Y, polygenic risk scores are considered high risk if they are in the top 2%, or in other words, out of every 100 people, the polygenic score would need to be higher than at least 98 people. Polygenic risk scores in the top 2% are associated with greater than a 3.5-fold risk of developing disease Y, according to existing data. These results do not indicate that you have disease Y now or will definitely develop disease Y in the future. There are still steps you and your healthcare team can take to prevent development of the disease or diagnose and treat it early.</td></tr>
</table>

- How did you feel looking at this report?

- How do you feel you understood the information presented to you in this format?

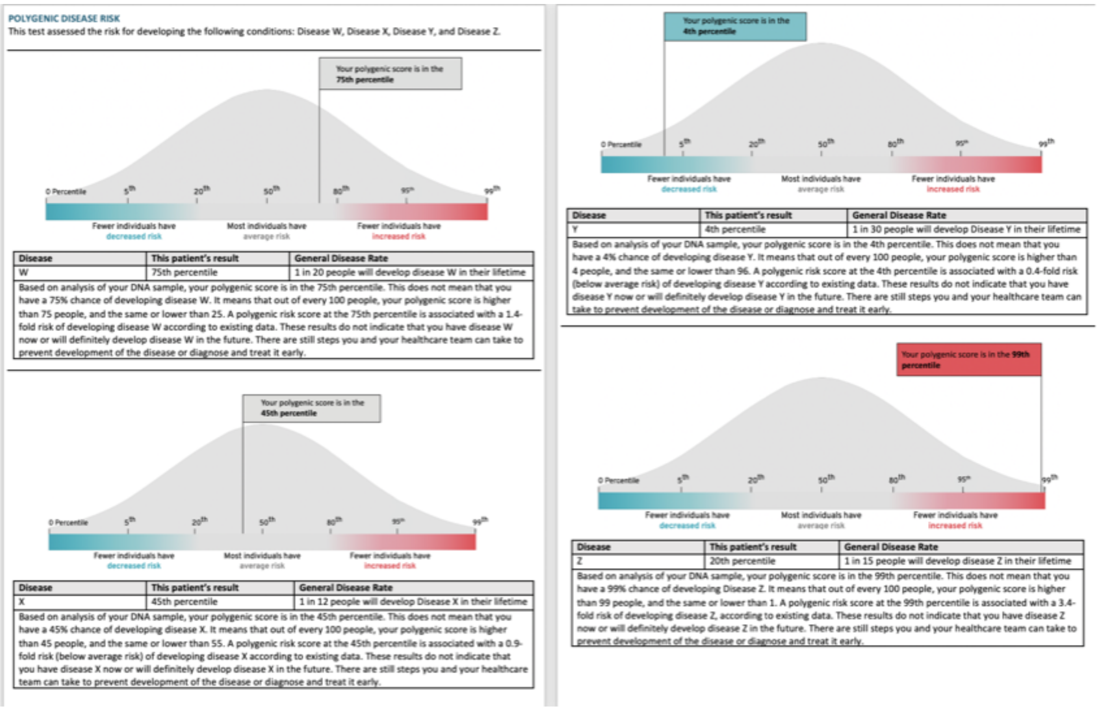

Now I will show you one last report with multiple polygenic risk scores in a different format. Please let me know when you are ready to discuss. Like in the previous report, the limitations section is not provided, but is identical to the previous limitations.

Physician Interview Guide eMERGE IV MGB

Italicized text is not to be read.

Hello, Dr. ________. Thank you for agreeing to take part in our interview study. My name is _____ and I am a research coordinator from Mass General Brigham Personalized Medicine. How are you today?

As a reminder, we are conducting a research study. The purpose of this interview is to hear your thoughts about the design of polygenic risk score reports for part of a larger research study. There are no right or wrong answers to these questions. This interview will be recorded and then transcribed and analyzed. All identifying information will be removed and all of the analyzed data will remain anonymous. Is it okay if I begin the recording now?

This interview will last about 60 minutes. For participating in this interview, we will send you a $200 check as a thank you for your time. Your participation in this interview is completely voluntary and if at any point you wish to stop the interview, you are welcome to do so. Additionally, if there is a question you would rather not answer, just let me know. Do you have any questions before we get started?

First, I'd like to ask you about your prior experiences with genetics

- How would you describe your level of genetic knowledge?
- Had you heard of polygenic risk scores before reading about this study?
  - **Prompt**: If so, in what context?
- Tell me about your experience with genetics in your practice?

Polygenic risk describes the chance of developing certain health conditions based on adding up the small effects of a large number of genetic variants across the genome. A typical polygenic risk score can be calculated from hundreds or even millions of genetic markers in a patient's DNA. Studies establish that these scores are correlated with risk for a given disease.

You can get these scores for many conditions, such as coronary artery disease and type 2 diabetes. One active area of research is whether polygenic scores should be used to help doctors and patients make decisions about, for example, statin therapy to prevent coronary artery disease or early mammography to screen for breast cancer. In this interview, we're going to focus on a polygenic risk score for prostate cancer.

- In your practice, what is your general approach to prostate cancer screening?
  - Prompt: How do you use PSA testing in your practice, if at all?

Other risk factors
Show slide below.

Other factors that contribute to prostate cancer risk

Family History Having a father or brother with prostate cancer more than doubles a man's risk of developing this disease.

Monogenic variants Variants in BRCA1/2 increase the odds that a patient will develop prostate cancer, but only affect a small percentage of cases overall

Other risk factors

- Some research has linked smoking to a possible small increased risk of dying from prostate cancer
- Prostate cancer develops more often in African-American men and in Caribbean men of African ancestry than in men of other races.

A polygenic risk score is just one risk factor. For prostate cancer, here are some other risk factors. For now, please imagine that you have this information listed for this patient, such that you can consider it along with the polygenic risk score report.

Reactions to Dichotomous vs. Continuous reports
Now I will show you one example of a polygenic risk score report. Imagine that you get a report for one of your patients who participated in a research study on this topic. The overall report would look something like this. *Show slide below.* We're going to zoom in on all the text shortly, I don't expect you to read it here. Throughout this interview we will show you a few different versions of this report. Of note, it's just the Main Result and Explanation Text that will change between the reports.

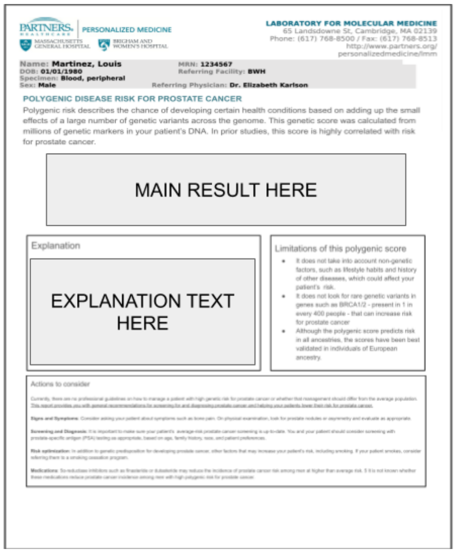
PARTNERS. HEALTHCARE | PERSONALIZED MEDICINE
MASSACHUSETTS GENERAL HOSPITAL   BRIGHAM AND WOMEN'S HOSPITAL

LABORATORY FOR MOLECULAR MEDICINE
65 Landsdowne St, Cambridge, MA 02139
Phone: (617) 768-8500 / Fax: (617) 768-8513
http://www.partners.org/personalizedmedicine/lmm

Name: Martinez, Louis   MRN: 1234567
DOB: 01/01/1980   Referring Facility: BWH
Specimen: Blood, peripheral
Sex: Male   Referring Physician: Dr. Elizabeth Karlson

POLYGENIC DISEASE RISK FOR PROSTATE CANCER
Polygenic risk describes the chance of developing certain health conditions based on adding up the small effects of a large number of genetic variants across the genome. This genetic score was calculated from millions of genetic markers in your patient's DNA. In prior studies, this score is highly correlated with risk for prostate cancer.

MAIN RESULT HERE

Explanation
EXPLANATION TEXT HERE

Limitations of this polygenic score
- It does not take into account non-genetic factors, such as lifestyle habits and history of other diseases, which could affect your patient's risk.
- It does not look for rare genetic variants in genes such as BRCA1/2 - present in 1 in every 400 people - that can increase risk for prostate cancer
- Although the polygenic score predicts risk in all ancestries, the scores have been best validated in individuals of European ancestry.

Actions to consider
[illegible]

The header information looks like this. Please let me know when you have had a chance to review. *Show slide below.*

PARTNERS HEALTHCARE | PERSONALIZED MEDICINE

MASSACHUSETTS GENERAL HOSPITAL   BRIGHAM AND WOMEN'S HOSPITAL

LABORATORY FOR MOLECULAR MEDICINE
65 Landsdowne St, Cambridge, MA 02139
Phone: (617) 768-8500 / Fax: (617) 768-8513
http://www.partners.org/personalizedmedicine/lmm

Name: **Martinez, Louis**	MRN: **1234567**
DOB: **01/01/1980**	Referring Facility: **BWH**
Specimen: **Blood, peripheral**	
Sex: **Male**	Referring Physician: **Dr. Elizabeth Karlson**

POLYGENIC DISEASE RISK FOR PROSTATE CANCER

Polygenic risk describes the chance of developing certain health conditions based on adding up the small effects of a large number of genetic variants across the genome. This genetic score was calculated from millions of genetic markers in your patient's DNA. In prior studies, this score is highly correlated with risk for prostate cancer.

Next let's look at the "Actions to Consider" section. Please let me know when you have had a chance to review. *Show slide below.*

Actions to consider

Currently, there are no professional guidelines on how to manage a patient with high genetic risk for prostate cancer or whether that management should differ from the average population. This report provides you with general recommendations for screening for and diagnosing prostate cancer and helping your patients lower their risk for prostate cancer.

Signs and Symptoms: Consider asking your patient about symptoms such as bone pain. On physical examination, look for prostate nodules or asymmetry and evaluate as appropriate.

Screening and Diagnosis: It is important to make sure your patient's average-risk prostate cancer screening is up-to-date. You and your patient should consider screening with prostate-specific antigen (PSA) testing as appropriate, based on age, family history, race, and patient preferences.

Risk optimization: In addition to genetic predisposition for developing prostate cancer, other factors that may increase your patient's risk, including smoking. If your patient smokes, consider referring them to a smoking cessation program.

Medications: 5α-reductase inhibitors such as finasteride or dutasteride may reduce the incidence of prostate cancer risk among men at higher than average risk. 5 It is not known whether these medications reduce prostate cancer incidence among men with high polygenic risk for prostate cancer.

Now let's look at the actual results section. *(Interviewer shares screen of increased risk dichotomous/continuous report, randomized to which is shown first).* Please review and let me know when you are ready for my questions.

If doing dichotomous report first, show the below:

Polygenic score indicates a high risk for prostate cancer

Explanation

Your patient's polygenic risk score has been associated with a **high polygenic risk** for prostate cancer. Out of every 100 people, your patient's polygenic score is higher than at least 95 people. Those at high risk (top 5%) have increased odds of developing prostate cancer that varies by ancestry:

- 1.5-2.5 times for African ancestry
- 2.2-4.1 times for Asian ancestry
- 3.1-4.5 times for European ancestry
- 1.9-3.2 times for Latino/a/x ancestry

The prevalence of prostate cancer in the general male population is 1 in 8.

Limitations of this polygenic score

- It does not take into account non-genetic factors, such as lifestyle habits and history of other diseases, which could affect your patient's risk.
- It does not look for rare genetic variants in genes such as BRCA1/2 - present in 1 in every 400 people - that can increase risk for prostate cancer
- Although the polygenic score predicts risk in all ancestries, the scores have been best validated in individuals of European ancestry.

Reaction to first increased risk polygenic risk score report

- How would you interpret this?
    - **Prompt:** Do you feel comfortable with interpreting odds?
- Are there any immediate questions you have while looking at this report? (If question includes one of our questions below, ask them to try to answer themselves)
- What is your understanding of the limitations section?
    - **Prompt:** What is your understanding of the last bullet of the limitations?
- If you had a patient of non-European ancestry, how would you talk about this report with them?
    - **Prompt:** How would you use the bullets in the Explanation section?
- Would you feel comfortable discussing this with your patient?
    - **Prompt**: How would you communicate this to your patient?
    - **Prompt**: What questions do you think they would have?
    - **Prompt:** How do you think they would respond to receiving this information?
- Would you consider any of the listed actions?
    - **Prompt:** Would this differ depending on whether your patient had any of the other risk factors?

Now I am going to show you a different risk report. Please note that the limitations have not changed. *Show side below.*

Polygenic score does not indicate a high risk for prostate cancer

Explanation

Your patient's polygenic risk score has not been associated with a high polygenic risk for prostate cancer. This means they are not classified as at high risk, which is defined here as within the top 5% of risk. Those at high risk (top 5%) have increased odds of developing prostate cancer that varies by ancestry:

- 1.5-2.5 times for African ancestry
- 2.2-4.1 times for Asian ancestry
- 3.1-4.5 times for European ancestry
- 1.9-3.2 times for Latino/a/x ancestry

The prevalence of prostate cancer in the general male population is 1 in 8.

Limitations of this polygenic score

- It does not take into account non-genetic factors, such as lifestyle habits and history of other diseases, which could affect your patient's risk.
- It does not look for rare genetic variants in genes such as BRCA1/2 - present in 1 in every 400 people - that can increase risk for prostate cancer
- Although the polygenic score predicts risk in all ancestries, the scores have been best validated in individuals of European ancestry.

Reaction to average risk polygenic risk score report

- Now that you've seen this version of this report, what changes in terms of your potential interpretation, questions you might have, your comfort level discussing this with your patient, or the actions you would consider?
  - **Prompt:** How do you interpret the risk for this patient?

EITHER *[For continuous only]*

- Does the absence of a suggested risk threshold affect the utility of this report from your perspective?
- Would you prefer that the report define the terms "not identified as at high risk" vs. "high risk" as well as showing the risk on a continuous scale?

OR *[For dichotomous only]*

- I've shown you both the high risk and not identified as at high risk report. There has to be some threshold chosen to separate these two categories. What factors do you think should go into the decision about where that line is drawn?
  - **Prompt:** How do you feel about the cut-off we have chosen?
  - **Prompt:** How do you feel about the patients at e.g. the 90th %ile receiving a not identified as at high risk report?

Now I am going to show you a risk report that uses a different way of displaying the results. Please note that the limitations have not changed. Please let me know when you are ready for my questions. *Show side below.*

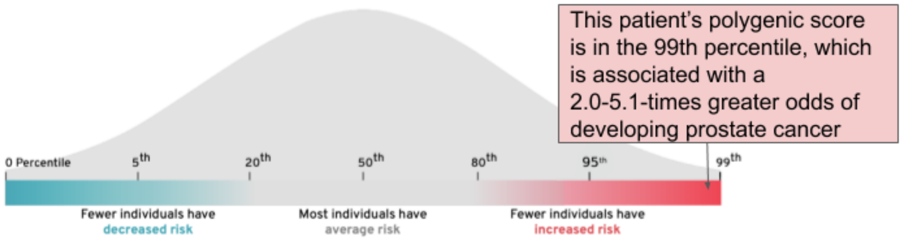

Explanation

Your patient's polygenic risk score is in the 99th percentile, meaning that out of every 100 people, their score is higher than 99 people, and the same or lower than 1. This value for polygenic risk was associated with increased odds of developing prostate cancer that varies by ancestry:

- 2.0-3.0 times for African ancestry
- 2.9-4.9 times for Asian ancestry
- 3.6-5.1 times for European ancestry
- 2.4-3.9 times for Latino/a/x ancestry

The prevalence of prostate cancer in the general male population is 1 in 8.

Limitations of this polygenic score

- It does not take into account non-genetic factors, such as lifestyle habits and history of other diseases, which could affect your patient's risk.
- It does not look for rare genetic variants in genes such as BRCA1/2 - present in 1 in every 400 people - that can increase risk for prostate cancer
- Although the polygenic score predicts risk in all ancestries, the scores have been best validated in individuals of European ancestry.

Reaction to second increased risk polygenic risk score report

- Now that you've seen this version of this report, what changes in terms of your potential interpretation, questions you might have, your comfort level discussing this with your patient, or the actions you would consider?
    - **Prompt:** How do you interpret the risk for this patient?

Now I am going to show you another report. Please note that the limitations have not changed. *Show side below.*

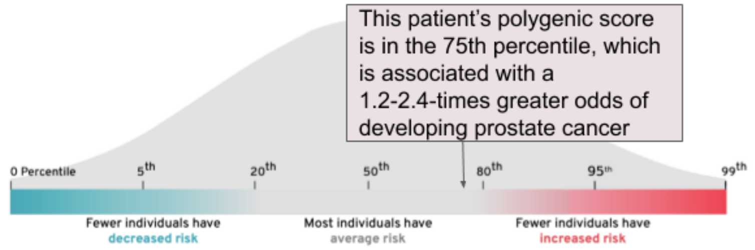

Explanation

Your patient's polygenic risk score is in the 75th percentile, meaning that out of every 100 people, their score is higher than 75 people, and the same or lower than 25. This value for polygenic risk was associated with increased odds of developing prostate cancer that varies by ancestry:

- 1.2-1.8 times for African ancestry
- 1.5-2.1 times for Asian ancestry
- 1.8-2.4 times for European ancestry
- 1.4-2.2 times for Latino/a/x ancestry

The prevalence of prostate cancer in the general male population is 1 in 8.

Limitations of this polygenic score

- It does not take into account non-genetic factors, such as lifestyle habits and history of other diseases, which could affect your patient's risk.
- It does not look for rare genetic variants in genes such as BRCA1/2 - present in 1 in every 400 people - that can increase risk for prostate cancer
- Although the polygenic score predicts risk in all ancestries, the scores have been best validated in individuals of European ancestry.

Reaction to second lower risk polygenic risk score report

- Now that you've seen this version of this report, what changes in terms of your potential interpretation, questions you might have, your comfort level discussing this with your patient, or the actions you would consider?
  - How do you interpret the risk for this patient?

EITHER *[For continuous only]*

- Does the absence of a suggested risk threshold affect the utility of this report from your perspective?
- Would you prefer that the report define the terms "not identified as at high risk" vs. "high risk" as well as showing the risk on a continuous scale?

OR *[For dichotomous only]*

- I've shown you both the high risk and not identified as at high risk report. There has to be some threshold chosen to separate these two categories. What factors do you think should go into the decision about where that line is drawn?
  - **Prompt:** How do you feel about the cut-off we have chosen?
  - **Prompt:** How do you feel about the patients at e.g. the 90th %ile receiving a not identified as at high risk report?

Preference between dichotomous and continuous report

- Between the report that used the text box with a dichotomous result (high or average) or that used a continuous scale graphic, which would you prefer to receive, and why?
  - **Prompt**: Is one version easier for you to understand?
  - **Prompt:** Would you feel more comfortable discussing one version with your patient?

    - **Prompt:** If you chose the continuous scale, would you also have liked a defined high risk threshold?
    - **Prompt:** If you chose the dichotomous report, would you have liked to see their exact percentile and/or odds ratio?
- Do you have any suggestions for improvements to any of the reports you have seen?
- Could you imagine yourself integrating this type of report into your primary care practice? Why or why not?

Integrate risk factors, or present them separately?

Now I would like to ask about a different choice concerning reporting. So far, you were told that you would see other risk factors separately — family history, clinical risk factors, and the polygenic risk score report.

I'd like to show you how all this information might appear on one page. Please note that we are only able to show information like this if we define a "high risk" threshold:

Result: High risk for prostate cancer

Polygenic risk: High risk

This patient's polygenic risk score has been associated with a high polygenic risk for prostate cancer. Out of every 100 people, your patient's polygenic score is higher than at least 95 people. Those at high risk (top 5%) have increased odds of developing prostate cancer that varies by ancestry:

- 1.5-2.5 times for African ancestry
- 2.2-4.1 times for Asian ancestry
- 3.1-4.5 times for European ancestry
- 1.9-3.2 times for Latino/a/x ancestry

Family history: not identified as at high risk

This patient does not report a father or brother with prostate cancer. Having a father or brother with prostate cancer more than doubles a man's risk of developing this disease.

Monogenic results: negative

No pathogenic/likely pathogenic variants were identified in BRCA1/2. Such variants are known to increase the odds of an individual developing prostate cancer. See full report in Epic.

Clinical factors: not identified as at high risk

Risk factor	Present
Smoker	No
African-American or Caribbean men of African ancestry	No

- Does anything change in terms of your potential interpretation, questions you might have, your comfort level discussing this with your patient, or the actions you would consider?
- Do you have any suggestions for improvements to this report?

In some cases, it is possible to just see a combined risk score that incorporates these risk factors. I am going to show you a combined risk score report, using the graphic that you indicated you preferred. Please take a look and let me know when you are ready for my questions. Please note that the "Actions to consider" are the same as you saw previously.

Interviewer shows either:

Combined score indicates a **high** risk for prostate cancer

Explanation

Your patient's combined risk score has been associated with a **high risk** for prostate cancer. Those at high risk (top 5%) have increased odds of developing prostate cancer that varies by ancestry:

- 2.0-3.1 times for African ancestry
- 2.4-4.5 times for Asian ancestry
- 3.3-4.7 times for European ancestry
- 2.0-3.5 times for Latino/a/x ancestry

This combined risk score incorporates your patient's family history, polygenic risk score, cholesterol level, smoking status, and whether or not they identify as African American

The prevalence of prostate cancer in the general male population is 1 in 8.

Limitations of this combined score

- It does not look for rare genetic variants in genes such as BRCA1/2 - present in 1 in every 400 people - that can increase risk for prostate cancer
- Although the polygenic score predicts risk in all ancestries, the scores have been best validated in individuals of European ancestry.
- Information your or your patient reported on their family history, cholesterol level and smoking status are assumed to have been accurately stated

Or:

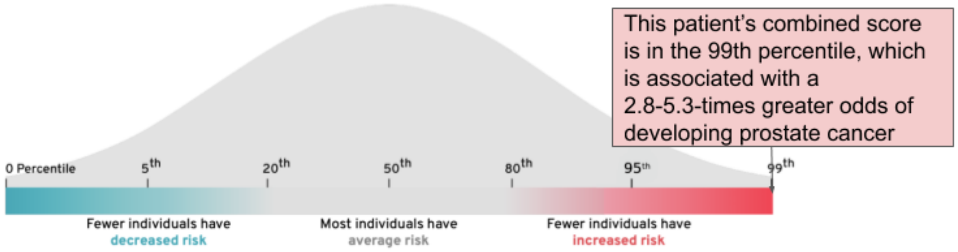

Explanation

Your patient's combined risk score is in the 99th percentile, meaning that out of every 100 people, their score is higher than 99 people, and the same or lower than 1. This value for combined risk score was associated with increased odds of developing prostate cancer that varies by ancestry:

- 2.9-3.9 times for African ancestry
- 2.9-4.9 times for Asian ancestry
- 3.8-5.3 times for European ancestry
- 2.8-4.1 times for Latino/a/x ancestry

This combined risk score incorporates your patient's family history, polygenic risk score, cholesterol level, smoking status, and whether or not they identify as African American.
The prevalence of prostate cancer in the general male population is 1 in 8.

Limitations of this combined score

- It does not look for rare genetic variants in genes such as BRCA1/2 - present in 1 in every 400 people - that can increase risk for prostate cancer
- Although the polygenic score predicts risk in all ancestries, the scores have been best validated in individuals of European ancestry.
- Information your or your patient reported on their family history, cholesterol level and smoking status are assumed to have been accurately stated

- Would you prefer to see a) just the polygenic report and other risk factors (as we considered before), b) just this combined score report, or c) the combined score report along with all the risk factors listed separately? Why?
    - **Prompt**: Would you show your patient one part of the report over another?
- The reports we have seen do not include absolute risk information, such as a patient's 5-year risk of developing prostate cancer. If you had access to this type of information, would it change how you would talk about the report with your patient?
    - **Prompt:** How do you tend to talk about disease risk with your patients?

Combined reports with multiple PRS

It is possible for polygenic risk scores to be reported simultaneously for multiple conditions, perhaps at least a dozen on the same report. I'm not going to show you what that report might look like.

- Do you have any additional benefits or concerns about a report covering multiple polygenic risk scores at once?
- Do you have points that we've discussed so far today that seem particularly relevant here, for example communicating results to your patient?
- In this context, do you see additional benefits or disadvantages to the choice of graphic that we considered earlier?

Wrap-up questions

I would like to ask some wrap-up questions about the prospect of the use of these scores in primary care.

- What do you see as the barriers and benefits for integration of this type of report into primary care?
- Under what conditions would you refer to a genetic counselor?
- What provider education, if any, would you consider necessary and practical?
    - **Prompt:** Would you use talking Points if provided?
    - **Prompt:** How much education do you think is necessary?
    - **Prompt:** What channels of education do you think would be best suited?

Finally, I'd like to ask some basic demographic questions

- Where do you currently practice?

As a zoom poll, or via displaying questions on a slide:

- With what gender do you identify? Male/Female/Other/Prefer not to answer
- Which of these best describe you? Please select all that apply
    - African American/Black
    - Latinx/Hispanic
    - Native American/Alaska Native/First Nations
    - Native Hawaiian/Pacific Islander
    - East/Southeast Asian
    - Middle Eastern/North African/West Asian
    - Caucasian/White
    - None of these fully describe me
    - Prefer not to answer
- How many years have you been practicing? 0-3 / 4-9 / 10-19 / 20-29 / 30-39 / 40+
- How many sessions for seeing patients do you have per week? 1-2, 3-4, 5-7, 7+
- What percentage of your patients belong to a minority racial or ethnic group? <10% / 10-20% / 21-40% / 41%-50% / >50%

Thank you so much for taking the time out of your day to complete this interview, I greatly appreciate it. I am ending the recording now. *(end recording)*. In order to send the $200 check to you, I will need your address and your Social Security Number. We require your social security number to process the payment per hospital policy. We keep this information confidential and hold it to the same privacy standards as patient data. Could you please provide me with those? Thank you again for your participation and have a great day. Good bye.
